# Systems-Based Analyses of Brain Regions Functionally Impacted in Parkinson's Disease Reveals Underlying Causal Mechanisms

**DOI:** 10.1371/journal.pone.0102909

**Published:** 2014-08-29

**Authors:** Brigit E. Riley, Shyra J. Gardai, Dorothea Emig-Agius, Marina Bessarabova, Alexander E. Ivliev, Birgit Schüle, Jeff Alexander, William Wallace, Glenda M. Halliday, J. William Langston, Scott Braxton, Ted Yednock, Thomas Shaler, Jennifer A. Johnston

**Affiliations:** 1 Elan Pharmaceuticals, South San Francisco, California, United States of America; 2 Thomson Reuters, Carlsbad, California, United States of America; 3 The Parkinson's Institute, Sunnyvale, California, United States of America; 4 Neuroscience Research Australia and the University of New South Wales, Sydney, N.S.W., Australia; 5 SRI International, Menlo Park, California, United States of America; National Institutes of Health, United States of America

## Abstract

Detailed analysis of disease-affected tissue provides insight into molecular mechanisms contributing to pathogenesis. Substantia nigra, striatum, and cortex are functionally connected with increasing degrees of alpha-synuclein pathology in Parkinson's disease. We undertook functional and causal pathway analysis of gene expression and proteomic alterations in these three regions, and the data revealed pathways that correlated with disease progression. In addition, microarray and RNAseq experiments revealed previously unidentified causal changes related to oligodendrocyte function and synaptic vesicle release, and these and other changes were reflected across all brain regions. Importantly, subsets of these changes were replicated in Parkinson's disease blood; suggesting peripheral tissue may provide important avenues for understanding and measuring disease status and progression. Proteomic assessment revealed alterations in mitochondria and vesicular transport proteins that preceded gene expression changes indicating defects in translation and/or protein turnover. Our combined approach of proteomics, RNAseq and microarray analyses provides a comprehensive view of the molecular changes that accompany functional loss and alpha-synuclein pathology in Parkinson's disease, and may be instrumental to understand, diagnose and follow Parkinson's disease progression.

## Introduction

Parkinson's disease (PD) is the second most common neurodegenerative disorder, affecting 1–2% of the population over the age of 65. It is a chronic movement disorder caused by relentless degeneration of specific neuronal populations in the brain, most notably the dopamine-producing neurons of the substantia nigra, which help control voluntary movement [1]. While the causes of PD are not completely understood, many PD-related genetic loci and large numbers of disease-causing mutations have been identified [2], [3], that may ultimately provide clues to disease pathogenesis. In addition to mechanisms related to genetic factors, sporadic PD may result from environmental factors (such as toxins) that cumulatively damage dopamine-producing neurons. In individuals who have defects in pathways dealing with oxidative stress, mitochondrial dysfunction, the ubiquitin proteasome system, or the autophagy-lysosome pathway it is possible that there is an increased sensitivity to environmental factors [4].

To date, many PD-related genetic loci and several disease-causing mutations have been identified [2], [3]. However, because the functional consequences of genetic alterations in PD are not understood, and any functional pathway connections between the identified PD genes are not yet established, the molecular basis of PD pathogenesis remains elusive. While the mechanisms underlying PD initiation and progression still remains a mystery, PD-related pathology and its progressive spread, initially described by Braak [5], [6], are a hallmark of pathogenesis. The pathologic changes include the progressive appearance of alpha-synuclein deposits coinciding with neuronal loss in interconnected circuits of the brain, including regions such as the salivary gland [7]–[10] that are not thought to be directly connected to the substantia nigra.

Coordinated and integrated activity of different brain regions is required for a variety of cognitive and motor functions, and the topographical sequence of Lewy body distribution and deposition in ascending brain regions is well documented [5], [6]. More recently, functional connectivity has been studied by means of electro- (EEG) and magnetoencephalography (MEG) and patients with advanced PD demonstrate decreased functional connectivity [11], [12]. The results of these cross-sectional studies suggest that changes in functional connectivity evolve over the course of the disease and may suggest that the observed loss of functional connectivity and neuronal network alterations reflect clinically relevant changes. Currently there is no clear understanding on how functional and pathologically affected brain regions may be connected at the molecular level and what, if any similar defects exist. Identification of the molecular mechanisms underlying the disease may help to suggest how disease progresses, aid in identification of early biomarkers of disease onset prior to clinical manifestation, and lead to new targets for therapeutic intervention [13]. PD pathogenesis has been investigated in-depth, providing gene expression and genetic variation data for integration and analysis [14]–[16]. However, no study has used the same tissue to directly compare two methods for gene expression changes and related this to alteration to protein levels, or tried to identify mechanistic alterations common to brain regions connected by pathology.

“Systems” analysis is believed to help deconvolute complex biological responses involving hundreds or thousands of genes assayed by OMICs methods. Although systems-style approaches have been applied to PD tissue, most studies have used simple functional overview approaches resulting in the identification of differentially expressed genes (DEGs), or pathways. While these approaches expanded our understanding of disease related changes, they are not able to elucidate the complex interconnectivity of biological and pathological processes present within diseased tissue. These approaches are considered “low resolution” descriptive methods and don't deliver executable hypothesis for subsequent follow-up experiments.

In addition to our novel approach of synthesizing expression and proteome data, we employed an additional level of complex functional assessment that takes into account protein interactions connecting genes (or proteins) within the dataset or whole proteome, and identifies and ranks genes based on their biological relevance for the phenotype. The interaction-focused method is high resolution, as it offers a list of ranked proteins that can be experimentally validated. Importantly, this approach identifies topologically significant genes, which are often missed in expression profiling experiments, as key regulatory genes, like transcription factors or kinases, change expression only transiently and on a small scale. Topologically significant genes are complementary to DEGs and are important in the reconstruction and integration of pathways and networks responsible for a given phenotype [17]. We hypothesize that integrating expression changes into higher order networks will allow for the identification of pathways, which are temporarily and spatially dysregulated in PD.

Using this high-resolution approach of integrating topologically significant genes, substantia nigra, striatum, and cortex samples from normal donors and patients with neurodegeneration (PD, dementia with Lewy bodies (DLB) and Alzheimer's disease (AD)) were subjected to microarray, RNAseq, and proteome analysis in an attempt to identify causal pathways dysregulated specifically in PD. The use of three different techniques allowed for the identification of common pathways, independent of the technology as well as highlighting the advantages and unique findings specific to each of these techniques. Additionally, we used this OMICs analysis to compile a panel of 50 biomarkers, which are expressed in the brain and periphery that, in conjunction with prodromal analysis might provide a path for early PD diagnosis. To our knowledge this is the first example in the literature of a systematic analysis of individual brain regions using microarray, RNAseq, and proteomics, providing a blueprint for future studies aimed at understanding the complex pathological changes that occur in PD.

## Results

### Genes and pathways independently and commonly dysregulated in PD brain regions

**Figure 1:**
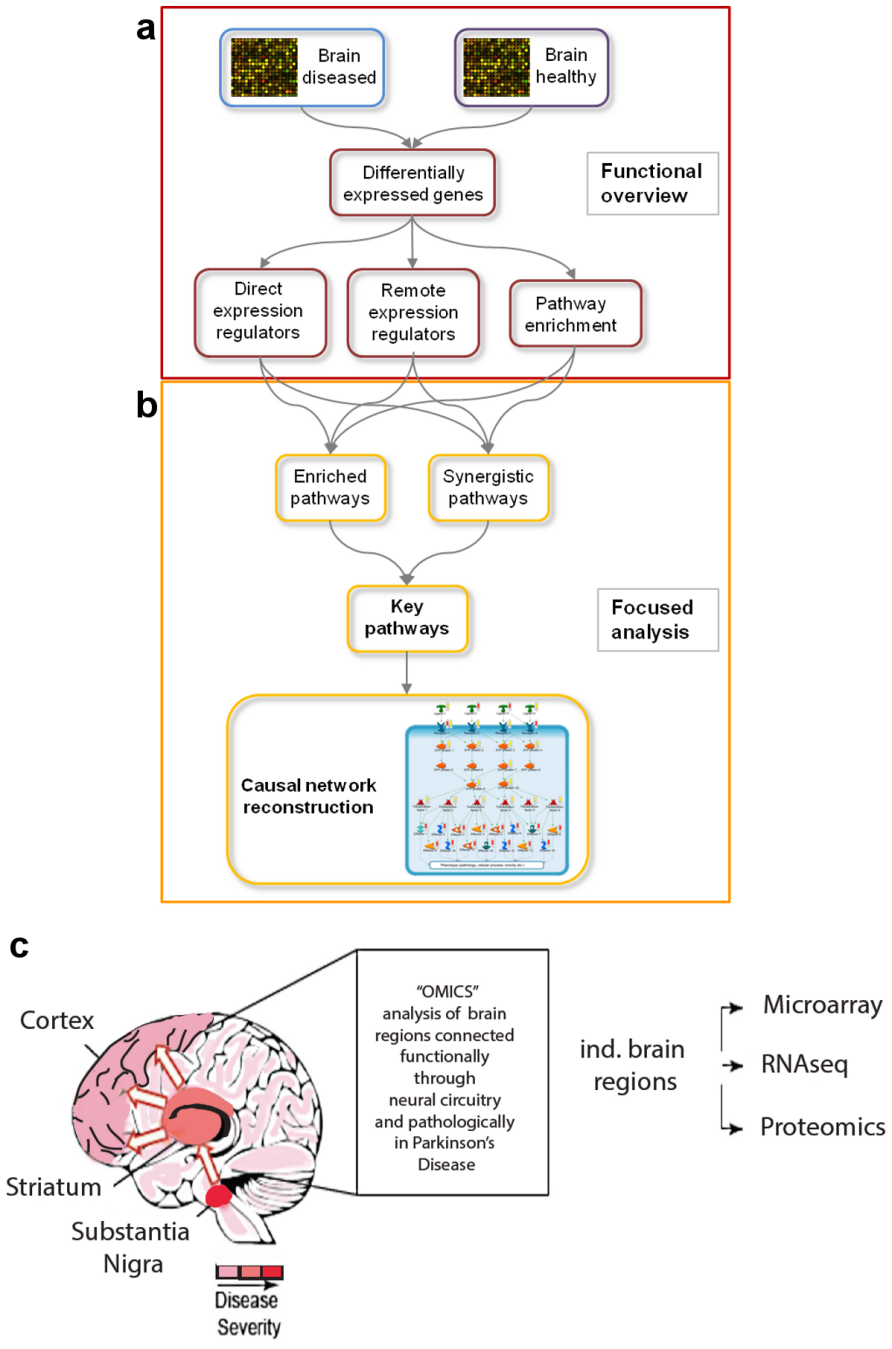
Overview of brain regions and methodology used in this study. (**a, b**) Overview of workflow for functional overview and focused analysis. (**a**) Expression data for healthy and diseased brain regions are statistically analyzed to obtain differentially expressed genes (DEGs). In the first part, the functional overview, the DEGs are used to identify expression regulators as well as pathways that are significantly enriched with DEGs. (**b**) In the second part, the focused analysis, pathways that are significantly enriched with expression regulators are combined with pathways that are significantly enriched with DEGs. Combining the two-pathway enrichment results leads to the identification of key pathways, which are the basis for the reconstruction of causal networks. (**c**) Cartoon representation of different brain regions used in the study, and the associated disease severity of each region denoted by gradations of red. Also shown is connectivity between the substantia nigra, striatum and cortex and the three methods used to interrogate the brain regions (microarray, RNAseq and proteomics).

**Figure 2:**
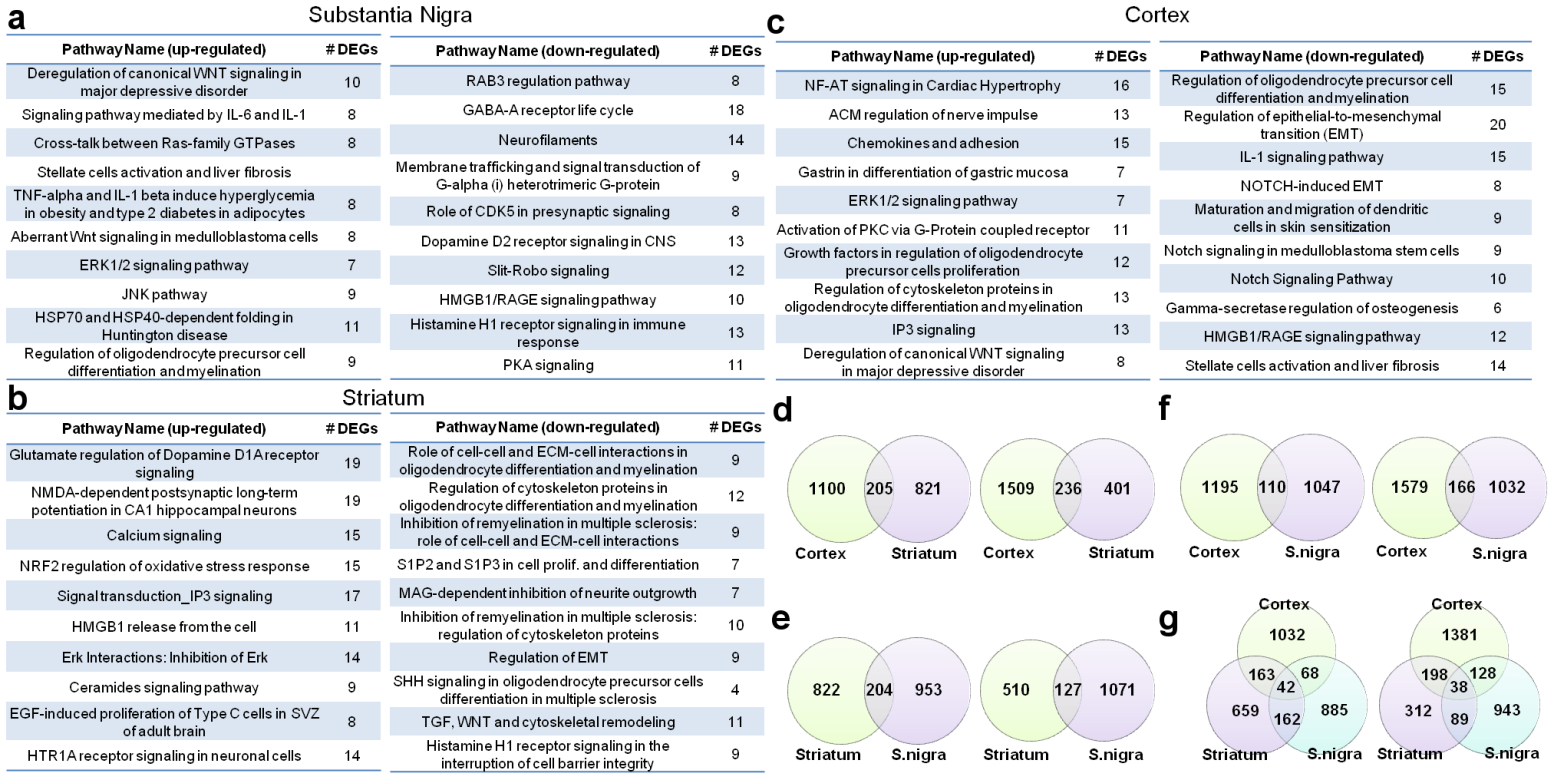
Identification of pathways dysregulated in PD-brain regions, and the overlap of differentially expressed genes (DEGs) between the PD-affected brain regions. The 10 key pathways most significantly enriched for DEGs in substantia nigra (**a**), striatum (**b**) and cortex (**c**) of PD brains compared to control as measured by microarray. Enrichments for upregulated genes are shown on the left, for downregulated genes on the right. The numbers of DEGs populating each pathway are denoted in the right columns (#DEGs). (**d**) Overlap between DEGs in PD cortex and striatum as measured by microarrays. The overlap of upregulated genes is shown on the left, the overlap of downregulated genes on the right. (**e**) Overlap between DEGs in striatum and substantia nigra (S.nigra) as measured by microarrays. The overlap of upregulated genes is shown on the left, the overlap of downregulated genes on the right. (**f**) Overlap between DEGs in PD cortex and substantia nigra (S. nigra) as measured by microarrays. The overlap of upregulated genes is shown on the left, the overlap of downregulated genes on the right. (**g**) Overlap between DEGs in PD cortex, striatum and substantia nigra (S. nigra) as measured by microarrays. The overlap of upregulated genes is shown on the left, the overlap of downregulated genes on the right.

To identify pathways dysregulated in brain regions shown to be functionally affected in PD, extracts from human brain samples were specifically prepared as described in Materials and Methods. Microarray expression analyses were performed in two main steps: 1) a *functional overview* providing insights into disease-associated genes and cellular processes (**Fig. 1a**), and 2) and a *focused analysis* to identify the most characteristic pathways associated with the disease as well as causal network reconstruction (**Fig. 1b**). Our samples, detailed in **Table S1**, were comprised of both males and females with an average age range of 60–70 years old, and with varying degrees of PD-associated pathology as verified by a certified pathologist. In general the substantia nigra samples had mild to moderate neuronal loss and depigmentation while the striatum and cortex samples appeared morphologically normal with little to no neuronal loss. We analyzed tissue from these three distinct brain regions thus capturing and comparing expression changes in brain regions exhibiting varying degrees of overt pathological changes (**Fig. 1c**, in **Table S1**).

To extend the functional overview analysis outlined in Figure 1a, the following steps were taken: 1. DEGs for each region were identified (**Table S2**), shown here are the top three up and down regulated genes from six unique controls and six unique PD donors for each region quantified by multiplexed quantitative gene analysis (QuantiGene from Panomics) (**Fig. S1** in File S1); 2. network-based analyses was performed to identify topologically significant genes, including key direct (one step away) (**Table S3**), and remote (multiple steps away) expression regulators (**Table S4**); 3. enrichment analysis was performed using pathway and other functional ontologies to provide insights into dysregulated cellular processes.

Following the functional overview, these results were utilized in the focused analysis to identify key pathways, i.e. pathways that are most reliably associated with the transcriptional changes found in PD. Key pathways served as the basis for causal network reconstruction and their creation is described in the methods. Full lists of key pathways dysregulated in the different brain regions as well as the DEGs and topologically significant genes populating the key pathways can be found in Tables S5, S6 and S7 respectively. Maps of key pathway dysregulated in the substantia nigra, striatum and cortex of PD brains versus control can be found in Figure 2a, b, c, respectively. While many top pathways identified using this more complex analysis were also found using simple individual component analysis, our approach revealed new dysregulated pathways including oligodendrocyte and synaptic vesicle misregulation. Importantly a number of the top dysregulated pathways were not associated with a general neurodegeneration response, as many of the pathways were unique to PD when compared with non-PD neurodegeneration samples (**Fig. S2** in File S1).

The top pathways dysregulated in the substantia nigra included upregulation of canonical WNT signaling, immune response mediated by IL-6 and IL-1β, and G-protein signaling cross-talk between the Ras-family of GTPases and as expected downregulation of dopamine D2 signaling and GABA receptor life cycle (**Fig. 2a** and **Fig. S3a** in File S1). In the striatum we found upregulated DEGs heavily populated the glutamate regulation of dopamine D1A receptor signaling, NMDA-dependent postsynaptic long-term potentiation, and Nrf2 regulation of oxidative stress pathways (**Fig. 2b** and **Fig. S3b** in File S1). Top pathways identified in the cortex that were not typically associated with PD or neurodegeneration included up-regulation of NFAT signaling, regulation of oligodendrocyte precursor cells, ACM regulation of nerve impulse, and chemokines and cell adhesion (**Fig. 2c** and **Fig. S3c** in File S1).

To identify common pathways affected in all brain regions we examined the overlap of DEGs between each region (**Fig. 2d, e, f**) and the combined union between all three-brain regions (**Fig. 2g**). The somewhat surprising extent of overlap, allowed us to construct and analyze causal network models. Causal network models are one of the most informative “high resolution” tools for analysis of gene expression data. Unlike the pre-built pathway maps that utilize cellular processes, which reflect general knowledge mined from the literature, condition-specific causal networks can be built to model molecular events in a particular dataset and disease state [18], [19].

### Causal networks linking functionally and pathologically connected brain regions

Understanding the nature of cause and effect is fundamental to all fields of scientific investigation. While DEGs and functional pathways affected in the brains of PD patients were identified, the complex nature of CNS (central nervous system) disorders makes it impossible to elucidate whether these changes directly contribute to pathology, or are simply a reflection of cell death, loss of critical regulatory signals, or are confounding effects of therapeutic treatment. To our knowledge this is the first attempt applying causal network models to deconvolve transcriptional changes present in the multifactorial environment of diseased tissue.

**Figure 3:**
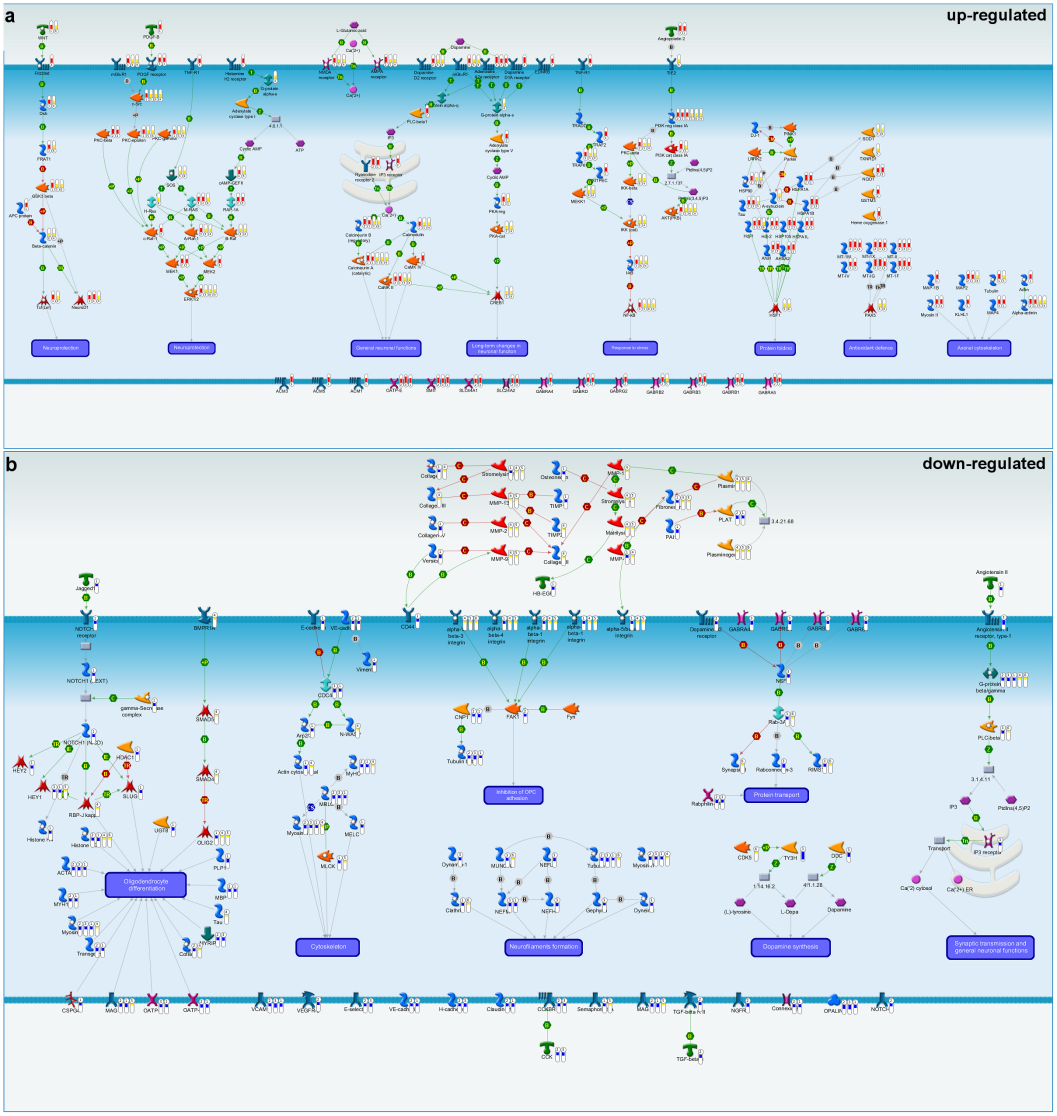
Creation of causal network models for PD brain regions reveals parallel yet distinct dysregulated pathways. (**a**) Integrated causal network model for upregulated genes in cortex (1), striatum (2), and substantia nigra (3) based on microarray data. Red thermometers represent upregulated genes. Yellow thermometers correspond to topologically significant genes in cortex (4), striatum (5), and substantia nigra (6). (**b)** Integrated causal network model for downregulated genes in cortex (1), striatum (2), and substantia nigra (3) based on microarray data. Blue thermometers represent downregulated genes. Yellow thermometers correspond to topologically significant genes in cortex (4), striatum (5), and substantia nigra (6).

To shed light on the causal processes underlying the expression changes observed in PD brain regions, the most dysregulated parts of key pathways were extracted and manually compiled into a new pathway map highlighting the most important PD-associated signaling cascades specific to each brain region (see methods). Causal models were built for both up and down regulated genes for each brain region (**Fig. S4** ((**a, b, c** upregulated and **d, e, f** downregulated) in File S1). These individual models were then integrated across the three brain regions to create a global causal map (**Fig. 3**). The united causal networks upregulated in PD brain regions included neuroprotection (WNT, PDGF and histamine H2), general neuronal functions (dopamine D2 and calcium signaling), long-term changes in neuronal function (dopamine D1A), response to stress (TNFα pathway), protein folding (heat-shock proteins), anti-oxidant defense (metallothionein family), and axonal cytoskeleton (actin and myosin) (**Fig. 3a**). Downregulated pathways include Notch signaling, Angiotensin II receptor, Oligodendrocyte function, Rab-3 signaling, and GABA receptors (**Fig. 3b**).

To pressure test the relevance and general applicability of our causal maps, we performed a meta-analysis using public PD microarray data recently compiled in the ParkDB database [20]. Some of the alterations in these previously unidentified causal pathways built from our data set were also present in these public data sets, indicating that our data set is comparable to the data sets previously reported on, however, our unique analysis highlights pathways previously hidden including oligodendrocyte differentiation, synaptic transmission, and anti-oxidant responses (**Fig. S5** in File S1).

To determine the applicability of PD causal networks to other synucleinopathies, microarray analysis of brains from patients with dementia with Lewy bodies (DLB) was performed. Patients with DLB have a build-up of alpha-synuclein containing Lewy bodies in the cortex rather than in the substantia nigra, therefore overlap analysis between PD cortex and DLB cortex as well as PD substantia nigra and DLB cortex was performed (**Fig. S6a** in File S2). Interestingly the overlap of up-regulated genes between the physiologically similar PD cortex and DLB cortex (8.4%, 109/1305) was substantially less than the overlap between the similarly diseased PD substantia nigra and DLB cortex (34.1%, 394/1157). Additionally we assessed DLB cortex for changes in causal pathways (**Fig. S6 b, c** in File S2) and found that many of the pathways altered in PD tissue were also affected in DLB cortex. These changes were specific for synucleinopathies, as microarray analysis of AD brains did not overlap at the DEG, pathway, or causal level with PD brains (**Fig. S2** in File S1 and **Fig. S7** in File S2). These data suggest disease-associated gene expression changes correlate more closely with PD pathology and alpha-synuclein deposition rather than physiological tissue similarity, although further studies are needed to confirm these findings.

### Different transcriptional techniques of PD tissue reveal similar alterations in causal networks

**Figure 4:**
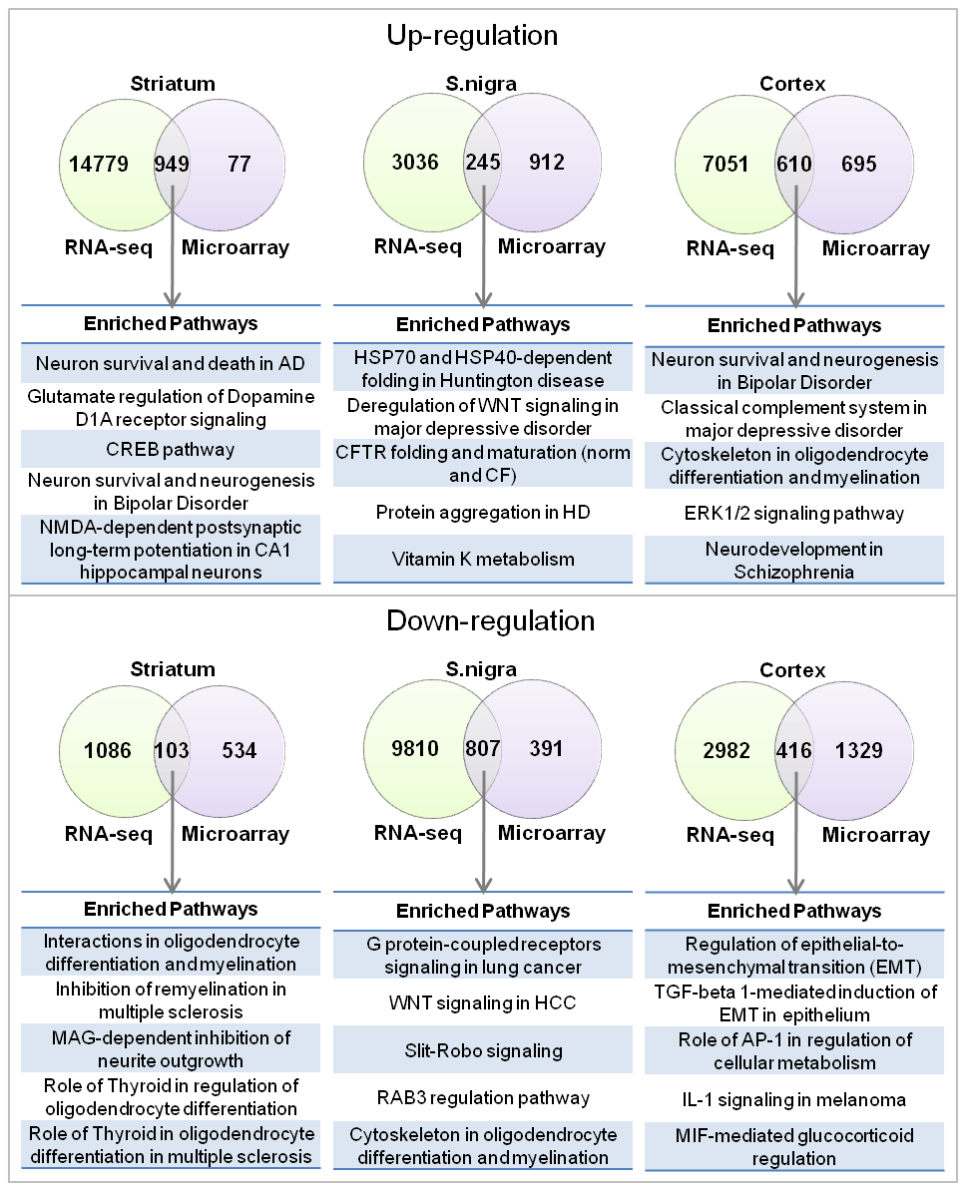
Overlap between pathways significantly enriched with differentially expressed genes identified by microarray and RNA-sequencing. (**a**) Overlap of pathways enriched with upregulated genes together with the five most significant overlapping pathways. (**b**) Overlap of pathways enriched with downregulated genes together with the five most significant overlapping pathways.

Currently there is a debate about the value of microarray versus RNAseq as a means to assess transcriptional changes. The technology to evaluate gene expression is constantly being refined, and while microarray analysis has been widely applied in biology, RNAseq technology may offer a less biased, more sensitive method to assess gene expression changes. While some direct comparisons have been performed [21]–[23], an assessment on PD tissue has not been executed. Therefore, a subset of normal and PD brain samples from substantia nigra, striatum and cortex were subjected to a 50 million read RNAseq analysis. The data was then processed to identify DEGs (**Table S8**). A functional overview, as described for our microarray data was also performed to identify enriched pathways (**Fig. S8** in File S2). Given the increased sensitivity reported for RNAseq technology it was not surprising that, when compared to the microarray data, the RNAseq analysis revealed many more significantly changed DEGs. However, the overlap of the DEGs from the two techniques was not complete, with individual brain regions influencing the extent of overlap (**Fig. 4a, b**). However, this difference in DEG detection was not reflected at the level of enriched pathways as the common shared pathways were similar to those identified by microarray (**Fig. 4a, b**) and this overlap was reflected as overlaps in the causal maps (**Fig. S9** in File S2).

### Protein alterations in PD brain are largely independent of transcriptional changes

**Figure 5:**
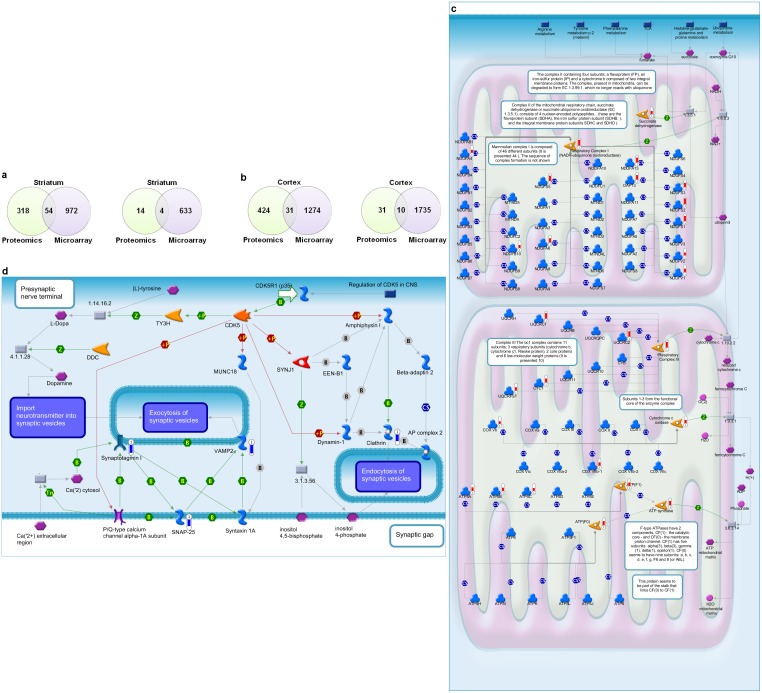
Protein alterations in PD brain are dominated by mitochondrial and lipid transport defects, and are largely independent of transcriptional changes. (**a**) Overlap between proteins and differentially expressed genes in striatum, as measured by mass spectrometry and microarray technologies. The overlap of upregulated proteins/genes is shown on the left, the overlap of downregulated proteins/genes on the right. (**b**) Overlap between proteins and differentially expressed genes in PD cortex, as measured by proteomics and microarray technologies. The overlap of upregulated proteins/genes is shown on the left, the overlap of downregulated proteins/genes on the right. (**c**) Oxidative phosphorylation pathway. The most significantly enriched upregulated pathway for PD cortex based on proteomics data. Red thermometers represent proteins with increased abundance. (**d**) Regulation of CDK5 in presynaptic signaling. The most significantly enriched downregulated pathway for PD cortex based on proteomics data. Blue thermometers represent proteins with decreased abundance.

The same samples used for microarray and RNA-sequencing were subjected to label-free quantitative mass spectrometry in order to quantify protein abundance changes in different PD brain regions (**Table S9**), and to understand the extent of overlap of protein changes with gene expression alterations. The overlap of proteomics with microarray at the level of DEGs represented only approximately 15% (54/372) of the significantly increased proteins in striatum (**Fig. 5a**), and approximately 7% (31/455) of increased proteins in the cortex (**Fig. 5b**). These data are highly suggestive that protein abundance changes are not reflected at the level of gene transcript in PD brain, and suggest that robust changes in protein degradation or protein translation occur in addition to changes in gene expression. As this could reflect sensitivity issues with microarray we also compared the proteomics data to the RNA-sequencing data and the results were similar (**Fig. S10a, b** in File S2). By mass spectrometry analysis, the top pathways with increased protein abundance in the striatum were cytoskeleton remodeling, glycolysis, and cell adhesion, and for the cortex they were oxidative phosphorylation, cytoskeleton remodeling and glycolysis (**Fig. S11a, b** in File S2). We validated increased levels via western blot (**Fig. S11c** in File S2). The primary defect identified in the cortex for the top dysregulated pathway, oxidative phosphorylation, were in complex 1 reflected by increased NDUF (NADH dehydrogenase ubiquinone) subunits (11/46 subunits with increased abundance), complex 3 (4/11 subunits with increased abundance) and ATPase synthases (**Fig. 5c**). Mitochondrial respiration defects have long been reported for PD patients [24]. However, recent studies also suggest deficiencies in mitochondrial quality control and mitochondrial fusion/fission could drive early pathogenesis and energy depletion [25].

We also detected several proteins with decreased abundance in the PD cortex, changes primarily driven by decreased levels of the vesicular transport proteins synaptotagmin 1, VAMP-2 and SNAP-25 (**Fig. 5d**). We validated reduced levels via western blot (**Fig. S11d** in File S2). Interestingly ubiquitin levels were decreased in both striatum and cortex likely reflecting a depletion of free ubiquitin pools. Future studies aimed at demonstrating the applicability of protein abundance changes in patient blood from early-stage PD patients will provide valuable insight and potentially new tools for early detection of PD prior to the onset of massive neuronal loss.

### Peripheral markers representative of causal changes identified from PD brain

**Figure 6:**
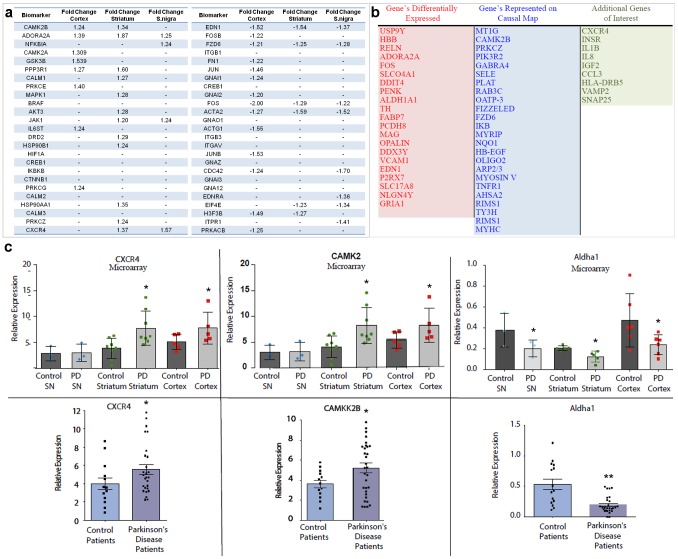
Tracking peripheral biomarkers identified from the causal mapping of PD brain. (**a**) High confidence biomarkers consistently identified for PD cortex, striatum, and substantia nigra (S. nigra) using microarray analysis. Upregulated biomarkers are shown on the left together with fold changes in the three brain regions, downregulated biomarkers on the right. (**b**) Functional biomarker panel that combines DEGs, high confidence biomarkers shown in (a) and genes from the causal map. (**c**) Assessment of biomarkers in brain (top panels)(substantia nigra, SN) and blood (bottom panels) from age-matched control and PD patients using QuantiGene technology from Panomics. * *p*≤0.05 and ** *p*≤0.01 as determined using a two-tailed unpaired t-test with Welch's correction.

CNS diseases are difficult to diagnose and neurodegeneration usually precedes observable clinical changes. Therefore, it is critical to identify peripheral markers that can be used as predictive biomarkers of CNS disease long before symptoms of degeneration arise. To that end we identified 50 potential biomarkers (25 up and 25 down) that were either present as DEGs in the brain or were key regulators in the causal pathways (**Fig. 6a**) Using these 50 potential biomarkers as a starting point we tailored our final biomarker list (54 genes) to include a subset of these genes in addition to DEGs with the most robust changes (**Fig. 6b** **and Table S10**). Using Panomics QuantiGene multiplex technology we validated the DEGs within the individual brain regions as well as key genes present along the causal pathway (**Fig. 6b**, **Fig. S1** in File S1) and measured gene changes in human blood isolated from nine age-matched control and 28 PD patients. As the biomarker lists were created based on genes changes in the CNS, many of the genes were not present in human blood. However, we were able to detect ten of these genes within human blood and a representative three are shown, CAMK2, CXCR4, and ALDHA1 with differential expression between control and PD correlating to what was found in PD brain (**Fig. 6c**). Recently we demonstrated that intra-cellular alpha-synuclein levels are elevated in the white blood cells of patients with PD [26]. To determine if alterations in our peripheral biomarkers were linked to elevated alpha-synuclein we performed a correlation analysis of intracellular alpha-synuclein levels quantified as mean fluorescent intensity with relative expression of four of our peripheral biomarkers (**Fig. S12** in File S2). Expression alterations in three of four of the biomarkers assessed (CAMK2B, CXCR4, ASHA2) correlated with alpha-synuclein levels. Overall, these data indicate that peripheral changes in PBMC's (peripheral blood mononuclear cells) are reflective of molecular alterations in the CNS and may act as part of a diagnostic panel for early detection of PD.

## Discussion

Elucidation of the causal events preceding the initiation and progression of disease pathogenesis, while extremely challenging, is one of the overarching goals of medical research. In an effort to reveal underlying molecular signatures common among unique brain regions affected during PD disease pathogenesis we undertook a systems-based approach utilizing data from microarray, RNAseq, and label-free quantitative mass spectrometry. Employing three analysis techniques on the same tissue samples allowed us to directly compare transcript and proteomic changes within disease tissue to derive underlying mechanistic insight provided by tissue analysis using three independent technologies.

Identification of DEGs and pathways using functional analysis, which has been employed by others, revealed changes previously described in PD brain tissue [15]. The bulk of previous studies have focused on transcriptional alterations, as assessed by microarray in the substantia nigra. When comparing these reports with our own findings, a high degree of overlap encompassing alterations in calcium signaling [27], Erk/MAPK pathway [28], and cytoskeletal changes [27] were observed, all of which are current areas for therapeutic intervention. Similarities with previous reports were also found in our cortex and striatal analysis, and included pathways such as chaperone response [29]. While many similarities were detected, our analysis pulled out unique changes, including down regulation of Rab-3 signaling in the substantia nigra and decrease in S1P2 and S1P3 (Sphingosine-1 phosphate receptor 2 and 3) in the striatum.

In line with what has been previously reported in other studies, the experimental data did not show large changes in numerous genes, and this partially explains why there has not been good convergence between the numerous PD microarray analyses [28]. Indeed if standardized pathway membership is used for comparison among different published microarray studies of PD tissue rather than ranked gene lists based on a fold change cutoff and p-value there is greater convergence between datasets [28]. The expression changes, as assessed by microarray analysis, were similar possibly due to the inherent heterogeneity in human disease, as this was true even when we increased sample size. Although the individual magnitude of changes may be minimal, the fact that genes work together to carry out functions, suggests that coherent changes in biologically meaningful sets of genes, may allow one to identify the biological processes that underlying these changes. Gene enrichment analysis performed here allowed for the detection of modest, but coordinate expression changes in functionally related genes. The importance and value of network modeling have been detailed by others [30]–[32] and have been used to identify underlying pathological processes in neurodegenerative and non-neurodegenerative diseases [33].

Additionally, common transcript changes across various brain regions were assessed using causal network analyses. In-depth investigation revealed several common molecular drivers up and downregulated in all three-brain regions. These changes were integrated and comprised our causal map. The causal signatures were shared between brain regions with differing pathology, and were not reflected in non-PD neurodegeneration. Although PD is clinically heterogeneous, we were able to isolate molecular commonalities shared between brain regions suggesting a unifying mechanism of PD could exist with parallel signatures engaged along the spectrum of neurodegeneration.

One of the most striking upregulated causal pathways consistently identified in all three-brain regions was metal homeostasis driven by robust expression of the metallothionein genes. These genes were increased in the substantia nigra GEO dataset [34] but only recently documented to be increased in a meta-analysis [35] of PD substantia nigra [34]. Multiple metallothionein genes (MT-1M, MT-1X, MT-II, and, MT-1G) were upregulated in the substantia nigra, striatum and cortex and PAX5 (paired-box 5 family) was identified as a topologically significant transcription factor linked to metallothionein activation. Recently metallothionein genes were shown to be dysregulated in lysosomal storage disorder (LSD) patient brain, and to function as markers of disease progression and therapeutic response [36] perhaps providing clues to the genetic link and underlying cause shared between PD and LSD [37].

A downregulated causal pathway identified from our study has implications for oligodendrocyte function/myelination and included transcript and proteomic changes in Olig2, MAG, PLP1, and MBP. While alterations in oligodendrocyte function have to our knowledge not been previously described, our analysis of the ParkDB confirmed that alterations in some of these genes have been detected but not highlighted before, again emphasizing the importance of our functional analysis. These molecular changes may explain the imaging alterations observed in the white matter of PD patients. For example, reports utilizing diffusion magnetic resonance imaging demonstrated widespread pathology of white matter in PD patients as well in patients with other synucleinopathies and this pathology was associated with executive dysfunction and changes in cognition [38], [39]. Determining whether these changes are associated with the lipid metabolism changes observed in the frontal cortex of PD patients [40], or is a reactionary change to the loss of dopamine still needs to be elucidated.

Although the causal pathways stemmed from microarray assessment, we also investigated transcriptional changes using RNAseq. To our knowledge this is the first report to subject the same tissue sample from normal and diseased patients to both microarray and RNAseq analysis. Not surprisingly the enhanced sensitivity of the RNAseq technique revealed more statistically significant dysregulated DEGs including alterations in POMC signaling, LRRK2 signaling in neurodegeneration, and multiple inflammatory pathways including IFN-gamma in TH2 cytokine-induced inflammation. While differences existed between these two analyses, comparison of DEG and pathway overlaps between microarray and RNAseq analyses highlighted pathways represented on the causal map.

The difference between microarray and RNAseq analysis may reside with the inherently low RIN (RNA integrity numbers) associated with human brain, on average significantly lower than other tissues [41]. A recent study suggested that, in addition to post mortem interval (PMI), RIN has a negative correlation with BMI (body mass index) and is a reproducible predictor of correlation between microarray data and follow up RT-PCR [41]. Microarray requires hybridization of transcripts with chips, however RNAseq techniques utilize fragmented RNA for sequencing and may not be as affected by low RINs. Moreover, microarray requires the transcripts to be present on the hybridization chip whereas RNAseq is able to assess all transcripts, and due to the high number of sequencing reads is able to pull out transcripts that may be present at a lower frequency.

Outside of the gene-expression signatures representing commonalities among unique brain regions affected by disease, we also observed protein homeostasis dysregulation. Robust alterations in protein levels in the least affected brain region, the cortex, suggested that massive cell death is not the driving force [42]. Although we did not analyze the substantia nigra by mass spectrometry, other groups have not reported overt changes in the levels of proteins in these pathways [43]–[45]. Recent studies estimate mRNA expression and protein levels have a modest correlation, with clusters of proteins exhibiting variance in levels independent of changes in mRNA expression, estimated at approximately 50% overlap [46]–[50]. In our case, the unequal overlap between transcript and protein was significantly affected by disease, with increased protein abundance observed for mitochondrial proteins and glycolytic enzymes in PD brain. In addition to defects in protein degradation and quality control, an intriguing possibility is that there are defects in protein translation driving the differences in protein abundance compared to gene expression in PD brain. Recent genetic linkage studies reported point mutations, albeit so far rare, in the translational initiator protein EIF4G1 [51]. In addition ribosomal proteins are over-represented in proteins associated with alpha-synuclein and DJ-1 [52] and there were defects in translation when the bacterial homolog of DJ-1 was deleted [53]. More recently additional quality control genes/mutations have been reported linked for Parkinsonism, including DNAJC6, DNAJC13, VPS35 and ATP6AP2 [54]. Future studies aimed at determining whether there are global defects in protein quality control and translation that correlates with disease severity in PD will be of interest.

While we did not detail alpha-synuclein, changes in our analysis were similar to other studies [20], [34], [55], we found that alpha-synuclein message levels were decreased in the substantia nigra (−1.38 fold) as assessed by microarray analysis and relatively unchanged in the other brain regions. Interestingly, upon performing sequential protein fractionation we found that alpha-synuclein levels were relatively constant in the soluble and Triton-X-100 detergent fractions, however there was an increase in alpha-synuclein, specifically high molecular weight alpha-synuclein in the insoluble fraction of all three PD brain regions, and the cortex of DLB patients (data not shown), with the most pronounced changes found, not surprisingly in the substantia nigra.

Perhaps the most important outcome of this study is our ability to detect changes in the periphery that were assembled from our molecular signatures identified from PD brain regions. Future studies using microarray analyses of fibroblasts or PBMCs isolated from normal donors or those affected with sporadic or familial PD overlaid onto these causal networks built from PD brains will be powerful to determine the feasibility of detecting early changes in the periphery prior to neurodegeneration and clinical manifestation of PD. While changes in the levels of peripheral proteins, e.g. derived from apoptosis of lymphocytes, have been reported to correlate with changes in PD neurons and may provide a source for proteins in plasma [56], we also hypothesize that proteins released from neurons will be found in blood plasma and provide a more direct source of potentially diagnostic biomarkers. This was recently reported in AD where the protein tau is released from affected neurons and detected peripherally. Proteins present in serum may exist in various forms; proteolytically processed fragments, normal cellular forms present as circulating immune complexes, or containing post-translational modifications. Overall, these data do represent a discovery sample set, and a new sample set will be needed for validation. Future studies aimed at measuring PD and control plasma to determine if similarities exist between cellular changes in brain homogenates and patient plasma will give direction to the development of suitable biomarkers or surrogate markers of PD for early diagnosis and treatment monitoring.

## Methods

### Tissue

Detailed description of the tissue used in our analysis can be found in **Table S1**.The human studies were performed following the study and protocol, that The Parkinson's Institutional Review Board approved, and all subjects gave written informed consent for this study. Tissues were generously provided by the Sydney Brain Bank at Neuroscience Research Australia, which is supported by the National Health and Medical Research Council of Australia (NHMRC), University of New South Wales, and Neuroscience Research Australia, (http://www.neura.edu.au/sydneybrainbank). Additional tissues were generously provided by the Human Brain and Spinal Fluid Resource Center, (11301 Wilshire Blvd. (127A) Building 212 Room 16 Los Angeles, CA 90073, http://brainbank.ucla.edu).

### RNA isolation

RNA isolation was performed by Expression Analysis (Durham, NC), and isolation was completed using the Qiagen miRNeasy Mini Kit. Briefly, the miRNeasy Mini Kit combines phenol/guanidine-based lysis of samples and silica membrane–based purification of total RNA. QIAzol Lysis Reagent is a monophasic solution of phenol and guanidine thiocyanate designed to facilitate lysis of tissues, to inhibit RNases, and also to remove most of the cellular DNA and proteins from the lysate by organic extraction. Tissue samples were homogenized in QIAzol Lysis Reagent. After addition of chloroform, the homogenate was separated into aqueous and organic phases by centrifugation. RNA partitions to the upper, aqueous phase, while DNA partitions to the interphase and proteins to the lower, organic phase or the interphase. The upper, aqueous phase was extracted, and ethanol was added to provide appropriate binding conditions for all RNA molecules from 18 nucleotides (nt) upwards. The sample was applied to the RNeasy Mini spin column, where the total RNA binds to the membrane and phenol and other contaminants were efficiently washed away. High quality RNA was then eluted in RNase-free water.

### Microarray

Expression Analysis (Durham, NC) performed the microarray according to their standard protocols. Briefly, prior to labeled target preparation, RNA samples were quantitated by spectrophotometry using a Nanodrop ND-8000 spectrophotometer, and assessed for RNA integrity using an Agilent 2100 BioAnalyzer or Caliper LabChip GX. RNA samples were converted to sense-stranded cDNA using the Ambion WT Expression Kit and subsequently labeled using the Affymetrix GeneChip WT Terminal Labeling Kit. The WT Expression kit is optimized for use with human, mouse and rat Affymetrix GeneChip Sense Target (ST) Arrays. Briefly, 200 ng of total RNA undergoes a reverse transcription step designed to exclude priming of ribosomal RNA. Primers are designed using a proprietary-oligonucleotide matching algorithm that avoids rRNA binding, thereby providing comprehensive coverage of the transcriptome while significantly reducing coverage of rRNA. This method also avoids the 3′ bias inherent in methods that prime exclusively with oligo-dT-based primers. The resulting sense-strand cDNA is next fragmented and labeled using uracil-DNA glycosylase (UDG) and apurinic/apyrimidinic endonuclease 1 (APE 1) which recognizes and fragments the cDNA at dUTP residues incorporated during the 2nd-cycle. Next, DNA is labeled by terminal deoxynucleotidyle transerase (TdT) using the Affymetrix DNA Labeling Reagent. Hybridization cocktail is then prepared using the Hybridization, Wash, and Stain kit (Affymetrix), applied to arrays, and incubated at 45°C for 16 hours. Following hybridization, arrays are washed and stained using standard Affymetrix procedures before scanning on the Affymetrix GeneChip Scanner and data extraction using Expression Console.

### RNAseq

Libraries are prepared for RNA-Seq as per the standard protocol; in brief sample libraries were prepared using the TruSeq RNA Sample Prep Kit (Illumina), including the use of Illumina in-line control spike-in transcripts. Prior to library preparation, RNA samples are quantitated by spectrophotometry using a Nanodrop ND-8000 spectrophotometer, and assessed for RNA integrity using an Agilent 2100 BioAnalyzer or Caliper LabChip GX. Library preparation begins with 500 ng of RNA in 50 µl of nuclease-free water, which is subjected to poly(A)+ purification using oligo-dT magnetic beads. After washing and elution, the polyadenylated RNA is fragmented to a median size of ∼150 bp and then used as a template for reverse transcription using random primers. The resulting single-stranded cDNA is converted to double-stranded cDNA, ends are repaired to create blunt ends, then a single A residue is added to the 3′ ends to create A-tailed molecules. Illumina indexed sequencing adapters are then ligated to the A-tailed double-stranded cDNA. A single index is used for each sample. The adapter-ligated cDNA is then subjected to PCR amplification for 15 cycles. This final library product is purified using AMPure beads (Beckman Coulter), quantified by qPCR (Kapa Biosystems), and its size distribution assessed using an Agilent 2100 BioAnalyzer or Caliper LabChip GX. Following quantitation, an aliquot of the library is normalized to 2 nM concentration and equal volumes of specific libraries are mixed to create multiplexed pools in preparation for sequencing.

### Panomics multiplex quantigene analysis

Gene probe ID's can be found in Table S10. Panomics (Santa Clara, CA) performed the quantigene analysis according to their standard protocols. Briefly samples were hybridized overnight at 54°C, washed 3×, hybridized with pre-amplifier at 50°C for 1 hour, washed 3× with 1× wash buffer, hybridized with Amplifier at 50°C for 1 hour, washed 3× with 1× wash buffer, hybridized with Label Probe at 50°C for 1 hour, washed 3× with 1× wash buffer, incubated with SAPE at room temperature for 30 minutes, read on a Luminex instrument. The sample input amount(s) for human brain samples was determined in a sample input optimization assay in which we determined that 400 ng/well of RNA was optimal for signal/noise ratio with out saturating housekeeping gene signals. Human universal RNA at 250 ng/well served as a positive control for the assay. RNase-free water in place of the RNA samples served as the background wells. The QuantiGene Plex 2.0 Human 57-plex panel data was exported from the BioPlex Instrument into Microsoft Excel and FI, FI-bkgd, Std dev, and % CV, for multiple analyte format was normalized again housekeeping genes, Gapdh, GusB, and Rplpo. For human blood analysis, 50 µl of flash frozen human whole blood samples were prepared according to the QuantiGene Sample Processing Kit for Blood Samples. Sample input optimization assay on a subset of the samples was run with the 57-plex panel. In brief lysate samples were loaded at 3 different amounts in singlet for input assay optimization. For the experimental assay, 12 µl per well for sample processing and all 46 human whole blood lysate samples were run in duplicate at 400 ng/well.

### Lysis conditions for proteomics analysis

Tissue samples were lysed in (450–600 µl) 20 mM HEPES (pH 7.2), 150 mM NaCl, 10% glycerol, 1% Triton X-100, 5 mg/ml NEM, 2% SDS and EDTA free complete protease inhibitors (Roche). Tissue samples were lysed using FastPrep Lysing Matrix D (MP Biomedicals) per manufacturer's instructions. FastPrep FP120 (Thermo) was used at speed 6.0, 40 s (×2); samples were then centrifuged at 14,000 rpm in a tabletop microcentrifuge for 10 minutes (×2). The supernatants were quantified using BCA (Pierce).

### Liquid chromatography- tandem mass spectrometry (LC-MS/MS)

The proteomics analysis of the trypsin-digested brain homogenates was performed by a label-free method combining LC-MS and LC-MS/MS techniques. The tryptic peptide mixtures were separated by capillary reversed-phase chromatography (0.3×150 mm C18-capillary column) at 8 microliters/min flow rate delivered from an Agilent Capillary 1100 pump (Agilent Technologies, Santa Clara, CA) employing a 2 hour-long binary gradient of 0% to 45% acetonitrile in water, both solvents modified with 0.1% formic acid. The effluent of the HPLC column was connected online to the electrospray ionization source of the mass spectrometer (Thermo LTQ-Orbitrap XL, Thermo Scientific, San Jose, CA). The primary MS1 mass spectra were acquired in the Orbitrap at resolving power of 60,000 (defined at m/z 400), while tandem mass spectra were acquired in the linear ion trap (LTQ) at a rate of five tandem mass spectra during each Orbitrap scan.

Protein identification was performed by searching the acquired tandem mass spectra for each sample against a human protein-database using byOnic software (Protein Metrics, Inc.). The false discovery rate (FDR) was estimated concatenating the full reversed-sequence set of proteins with the protein sequence database, with a cutoff value of 1% at the peptide and protein level. Relative quantitative information was extracted from the MS1 scans acquired for each sample using Sieve software (Thermo Scientific). For the proteomics analyses, the reproducibility that we used has been reported previously in method-development papers using replicate analyses of identical samples [57]–[60]. In our study we find that for proteins with no differences in abundance the median % CV values are in the 10–20% range which is typical for the method and consistent with previously published methods.

### Microarray expression data

Multiple microarray platforms were used in the expression analysis of different brain regions. Cortex samples were hybridized to the Affymetrix Gene ST 1.0 platform with 5 samples for PD cortex, DLB cortex, and control cortex, respectively. 6 PD and 6 control striatum samples (from the putamen) were hybridized to the Affymetrix Gene ST 1.0 platform. 11 PD, 11 control, and 3 non-PD striatum (from the putamen) samples were hybridized to the Affymetrix HG U133 Plus 2.0 platform. 3 PD and 3 control substantia nigra samples were hybridized to the Affymetrix Gene ST 1.0 platform. 13 PD and 11 control substantia nigra samples were hybridized to the Affymetrix HG U133 Plus 2.0 platform. Cortex samples were augmented with a publicly available dataset from GEO (GSE28894) where samples were hybridized with the Illumina humanRef-8 v2.0 expression bead-chip array.

### ParkDB expression data

Pre-processed lists of differentially expressed genes for cortex and substantia nigra were downloaded from the ParkDB database [20]. The parameters for differential gene expression were set to 1.2 fold change and 0.05 adjusted p-value. The cortex dataset comprised samples from *superior frontal cortex* (ArrayExpress id E-GEOD-8397), *prefrontal cortex area 9* (ArrayExpress id E-GEOD-20168), and *cortex area* (ArrayExpress id E-GEOD-13035). The substantia nigra dataset comprised samples from *substantia nigra* (ArrayExpress id E-GEOD-7621), *substantia nigra pars compacta* (ArrayExpress id E-GEOD-7307), *medial substantia nigra* (ArrayExpress id E-GEOD-8397), and *lateral substantia nigra* (ArrayExpress id E-GEOD-8397).

### Statistical processing of expression data

Microarray expression data has been deposited to NCBI Gene Expression Omnibus [61], accession number, GSE54282. Microarray expression data for each brain region were normalized with the RMA algorithm included in R using a custom CDF file from the brain array database [62], [63]. If samples from different platforms were available, they were first normalized separately and then merged into one dataset using Entrez gene ids as common identifier. Batch effect removal was performed using the ComBat algorithm to remove technical variation between results of the two platforms [64]. Quality assessment of merged data was performed to ensure that no separation by platform was observed after batch effect removal. Differential gene expression was identified using an empirical Bayes moderated t-test available in the Bioconductor limma package [65]. Genes were defined as differentially expressed if their absolute fold change was above 1.2.

Proteomics expression data were pre-processed as described above under mass spectrometry, and differentially expressed proteins were defined using an absolute fold change cut-off of 1.2 and p-value threshold of 0.05. RNA-sequencing data were pre-processed by Expression Analysis (http://www.expressionanalysis.com) and read count estimates were calculated using the RSEM algorithm [66]. Differentially expressed genes were identified using the DEGseq R-package using a fold change threshold of 1.2 for consistency with microarray parameters [67]. Gene and protein lists provided were mapped to Entrez gene identifiers using the DAVID bioinformatics resource [68].

### Enrichment analysis

A total of seven proprietary and publicly available ontologies were extracted from the MetaBase resource [69]: 1) *Pathway maps* represent a portfolio of over 1,000 canonical, disease-specific and toxicity-specific pathways. The goal of each pathway map is to show the cellular response to particular stimulus. Pathway maps are manually curated based on scientific articles and typically contain multiple linear directional pathways involved in similar biological outcomes. 2) *Process networks* represent reconstructions of more general metabolic and signaling processes and are usually larger than pathway maps. 3) *Toxicity networks* are manually curated network reconstructions of toxicity mechanisms. 4) *Disease biomarkers* are based on the classification in Medical Subject Headings (MeSH) and each disease in the ontology is assigned a set of biomarker genes. 5)–7) *GO processes*, *GO molecular functions*, and *GO Localizations* are regularly updated ontologies developed by the Gene Ontology [70]. Each set of differentially expressed genes was tested for overlap with pathway maps. The significance of the overlap between an entity in the ontology and a list of differentially expressed genes was calculated using a hypergeometric test:
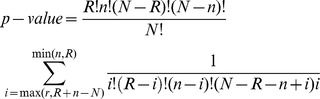

*R* corresponds to the number of genes contained in the list of differentially expressed genes. *n* corresponds to the number of genes contained a particular entity of the ontology. *N* represents the total number of genes contained in the ontology. *r* corresponds to the overlap between a particular entity in the ontology and the list of differentially expressed genes.

### Expression regulators

Expression regulators can be identified by mapping a set of differentially expressed genes onto a molecular interaction networks. Expression regulators correspond to those proteins that are responsible for the observed gene expression changes. All network-based analyses are based on the MetaBase resource, a manually curated interaction database [69].

- **Direct expression regulators.** Direct expression regulators correspond to genes that are directly interacting with an unexpectedly high number of differentially expressed genes. Direct regulators can be identified using an over connectivity test, which calculates the overlap of a protein's interactors and a list of differentially expressed genes [71]. We defined a protein as over connected, i.e. as a direct expression regulator, if the corresponding node in the interaction network had more direct interactions with differentially expressed genes than expected by chance. The significance of over connectivity was estimated using a hypergeometric test as described previously.
- **Remote expression regulators.** Remote expression regulators correspond to genes upstream of the differentially expressed genes. They are not required to directly interact with differentially expressed genes but are responsible for triggering signaling cascades that lead to the observed gene expression changes. Remote regulators can be identified using the Hidden Nodes algorithm [72]. The algorithm takes as input a molecular interaction network and a list of differentially expressed genes and creates a condition-specific sub-network by extracting the connections between the differentially expressed genes. Connections between differentially expressed genes are calculated based on shortest paths. Hidden Nodes evaluates the number of paths traversing a node in the condition-specific sub-network compared to the total number of paths traversing the same node in the input interaction network. Nodes associated with an unexpectedly high number of paths in the condition-specific sub-network are regarded remote expression regulators because of their topological importance in the network.

### Identification of key pathways

Key pathways correspond to those pathways that are most reliably associated with the observed transcriptional response and are required to be enriched with both differentially expressed genes and expression regulators. Key pathways were identified using the following calculations:

1. Pathway maps that are significantly enriched with differentially expressed genes were identified (the DEG set).
2. Pathway maps that are significantly enriched with expression regulators were identified (the regulator set).
3. Pathway maps that are significantly enriched with both differentially expressed genes and expression regulators were identified (the combination set).

To identify key pathways, the significance p-values resulting from the enrichment analyses in 1), 2) and 3) were compared: Pathway maps whose enrichment p-value in the combination set was *lower* than the p-values in the two sets separately were defined as “synergistically” enriched and correspond to key pathways.

### Causal network reconstruction

A causal network integrates prior biological knowledge with experimental data and describes the biological mechanisms underlying an observed phenotype. A causal network contains proteins and small molecules that are connected via directed molecular interactions. All interactions are supported by experimental evidence and are annotated with biological effects such as activation and inhibition. The structure of a causal network includes ligand-receptor interactions (triggers), signaling cascades via signal-transduction molecules, and transcription factors, which modulate the expression of downstream genes and lead to the observed phenotype. Here, causal networks were reconstructed using canonical pathway maps from the MetaBase resource, precisely the previously identified key pathways, since these maps contain experimentally validated interactions only and are structured the same way as a causal network.

Specifically, causal networks were reconstructed as follows: First, the differentially expressed genes and expression regulators were overlaid onto the key pathway maps. Differentially expressed genes and expression regulators were both considered affected in the phenotype. Second, affected genes were identified that correspond to ligands or receptors as they are regarded as triggers of the signaling cascades. Third, signal transduction pathways were selected that are 1) downstream of the affected triggers, 2) contain consecutive stretches of affected signal-transduction molecules, and 3) end at affected transcription factors. In addition to key pathway maps, groups of affected proteins were included in the causal network, where a group represents a well-defined functional module such as a protein complex.

### Key genes

Key genes represent genes that are important for the observed phenotype based on multiple sources of evidence. Each gene was evaluated based on the following criteria: 1) the gene is differentially expressed; 2) the gene is an expression regulator; 3) the gene is annotated with a GO biological process term that is enriched in the list of differentially expressed genes [73]; 4) the gene is annotated with a GO molecular function term that is enriched in the list of differentially expressed genes; 5) the gene is present on an enriched pathway map; 6) the gene is present on an enriched process network as stored in MetaBase; 7) the gene is present on an enriched toxicity network as stored in MetaBase; 8) the gene is present on a key pathway map; 9) the gene is present on the reconstructed causal network; and 10) the gene is expressed in whole blood. Whenever a gene fulfilled a requirement, it received a score of 1 in this particular category and 0 otherwise. Therefore, the maximum score a gene may receive is 10 and the minimum score is 0. Genes with high scores are regarded key genes and if expressed in blood they may serve as potential biomarkers for the phenotype.

## Supporting Information

Table S1 **Patient information.**
(XLSX)

Table S2 **DEG lists for microarray.**
(XLSX)

Table S3 **Direct expression regulators for microarray data.**
(XLSX)

Table S4 **Remote expression regulators for microarray data.**
(XLSX)

Table S5 **Key Pathways.**
(XLSX)

Table S6 **DEGs on Key Pathways.**
(XLSX)

Table S7 **Topologically Significant Genes on Key Pathways.**
(XLSX)

Table S8 **DEG lists for RNAseq.**
(XLSX)

Table S9 **Differentially changed protein lists for proteomics.**
(XLSX)

Table S10 **Panomics Gene Probe IDs.**
(PDF)

File S1 **Contains Figures S1–S5: Figure S1.** Validation of microarray using Panomics QuantiGene multiplex technology (denoted rt-PCR). **Figure S2.** Enriched pathway maps in PD and non-PD tissues. **Figure S3.** Key pathways for different brain regions. **Figure S4.** Causal network models of individual brain regions. **Figure S5.** Causal network models from different brain regions overlaid with ParkDB DEGs.
(DOC)

File S2 **Contains Figures S6–S12: Figure S6.** Comparison of PD cortex, DLB cortex, and PD substantia nigra. **Figure S7.** Integrated causal network models from different brain regions. **Figure S8.** Enriched pathway maps and DEG overlaps in different brain regions identified by RNA-sequencing analysis. **Figure S9.** RNA-sequencing expression data overlaid onto causal networks. **Figure S10.** Overlap between proteomics and RNA-sequencing data. **Figure S11.** Enriched pathways and western blot validation of proteomics data. **Figure S12.** Alpha-synuclein levels correlated with peripheral biomarker levels.
(DOC)
