# Correction: Systems-Based Analyses of Brain Regions Functionally Impacted in Parkinson's Disease Reveals Underlying Causal Mechanisms

**DOI:** 10.1371/journal.pone.0115081

**Published:** 2014-12-01

**Authors:** The PLOS ONE Staff

The sixth author’s name is spelled incorrectly. The correct name is: Birgitt Schüle.
