# Supplementary material for: Systems-Based Analyses of Brain Regions Functionally Impacted in Parkinson's Disease Reveals Underlying Causal Mechanisms
Source: PLoS One. 2014 Aug 29;9(8):e102909. doi: 10.1371/journal.pone.0102909 (PMC4149353; doi:10.1371/journal.pone.0102909)
Supplement: Table S10 — Panomics Gene Probe IDs. (PDF) [file pone.0102909.s010.pdf]

Supplementary Table S10: Panomics Gene Probe IDs

Gene	Accession	Gene	Accession	Gene	Accession
ACTR3	NM_005721	HBEGF	NM_001945	PENK	NM_001135690
ADORA2A	NM_000675	HLA-DRB5	NM_002125	PIK3R2	NM_005027
AHSA2	NM_152392	HPRT1	NM_000194	PLAT	NM_000930
ALDH1A1	NM_000689	IGF2	NM_000612	PPIB	NM_000942
CAMK2B	NM_001220	IL1B	NM_000576	PRKCZ	NM_002744
CCL3	NM_002983	IL8	NM_000584	RAB3C	NM_138453
CXCR4	NM_003467	INS	NM_000207	RELN	NM_005045
DDIT4	NM_019058	INSR	NM_000208	RIMS1	NM_014989
DDX3Y	NM_004660	MAG	NM_002361	RPLP0	NM_001002
EDN1	NM_001955	MT1G	NM_005950	SELE	NM_000450
FABP7	NM_001446	MYH16	NR_002147	SLC17A8	NM_139319
FOS	NM_005252	MYRIP	NM_015460	SLCO1A2	NM_134431
FZD1	NM_003505	NFKBIA	NM_020529	SLCO4A1	NM_016354
FZD6	NM_003506	NLGN4Y	NM_014893	SNAP25	NM_003081
GABRA4	NM_000809	NQO1	NM_000903	TH	NM_199292
GAPDH	NM_002046	OLIG2	NM_005806	TNFRSF1A	NM_001065
GRIA1	NM_000827	OPALIN	NM_033207	USP9Y	NM_004654
GUSB	NM_000181	P2RX7	NM_002562	VAMP2	NM_014232
HBB	NM_000518	PCDH8	NM_002590	VCAM1	NM_001078
