# Supplementary material for: Systems-Based Analyses of Brain Regions Functionally Impacted in Parkinson's Disease Reveals Underlying Causal Mechanisms
Source: PLoS One. 2014 Aug 29;9(8):e102909. doi: 10.1371/journal.pone.0102909 (PMC4149353; doi:10.1371/journal.pone.0102909)
Supplement: File S1 — Contains Figures S1–S5: Figure S1. Validation of microarray using Panomics QuantiGene multiplex technology (denoted rt-PCR). Figure S2. Enriched pathway maps in PD and non-PD tissues. Figure S3. Key pathways for different brain regions. Figure S4. Causal network models of individual brain regions. Figure S5. Causal network models from different brain regions overlaid with ParkDB DEGs. (DOC) [file pone.0102909.s011.doc]

**Figure S1**

**Substantia Nigra** *Microarray rt-PCR* **Striatum** *Microarray rt-PCR* **Cortex** *Microarray rt-PCR*

| MTIG | 2.02 | 2.7 | MTIG |  | 2.02 | 2.29 | DDX3Y |  | 3.39 | 2.71 |
| --- | --- | --- | --- | --- | --- | --- | --- | --- | --- | --- |
| DDX3Y | 2.53 | 1.36 | PENK |  | 2.04 | 1.72 | USP9Y |  | 2.64 | 2.48 |
| P2RX7 | 1.76 | 1.97 | ADORA2A |  | 1.87 | 1.15 | MT1G |  | 2.01 | 1.74 |
| TH | -3.47 | ‐2.46 | OPALIN |  | -1.91 | -1.78 | FOS |  | -1.99 | ‐2.04 |
| ALDH1A1 | ‐2.96 | ‐1.61 | MAG |  | -1.6 | -1.53 | MAG |  | -1.71 | ‐2.05 |
| OPALIN | ‐2.12 | ‐4.45 | FABP7 |  | ‐1.66 | ‐1.22 | OPALIN |  | -1.57 | ­2.24 |

**Supplementary Figure S1| Validation of microarray using Panomics QuantiGene multiplex technology**

**(denoted rt-PCR).** Validation of microarray data using 6 unique controls and Parkinson’s disease donors for each

region by Panomics QuantiGene multiplex technology (denoted rt-PCR). Fold change for three up-regulated and three down-regulated genes from microarray and rt-PCR analysis depicted here.


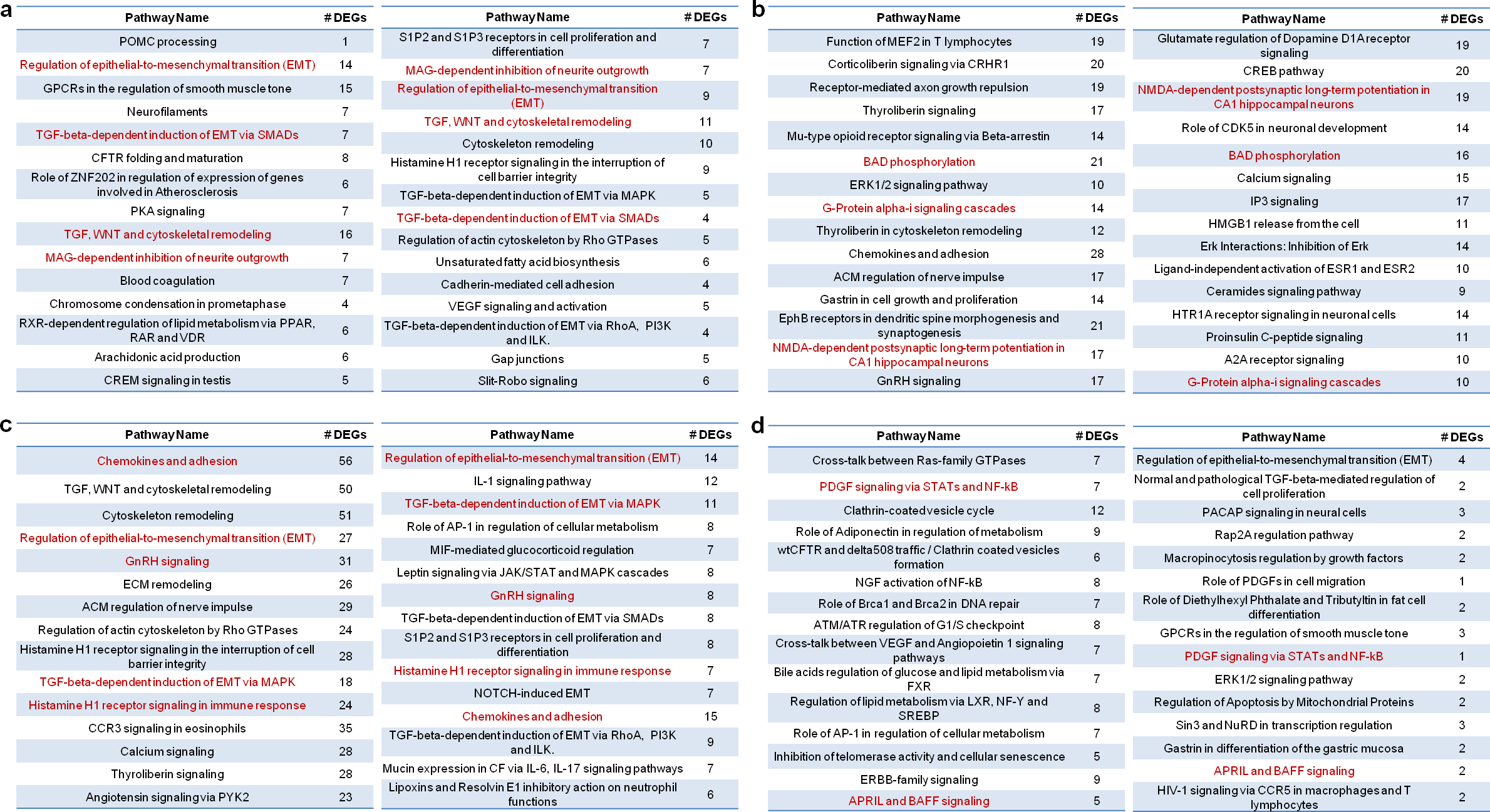
**Figure S2**

**Supplementary Figure S2 | Enriched pathway maps in PD and non-PD tissues.** (a) Top 15 pathways enriched with down-regulated genes in non-PD striatum on the left and in PD striatum to the right. (b) Top 15 pathways enriched with up-regulated genes in non-PD striatum on the left and in PD striatum to the right. (c) Top 15 pathways enriched with down-regulated genes in DLB cortex on the left and in PD cortex to the right. (d) Top 15 pathways enriched with up-regulated genes in DLB cortex on the left and in PD cortex to the right. Identical pathways for PD and non-PD tissues are highlighted in red. The number of DEGs on each pathway is shown in the #DEG columns.

**
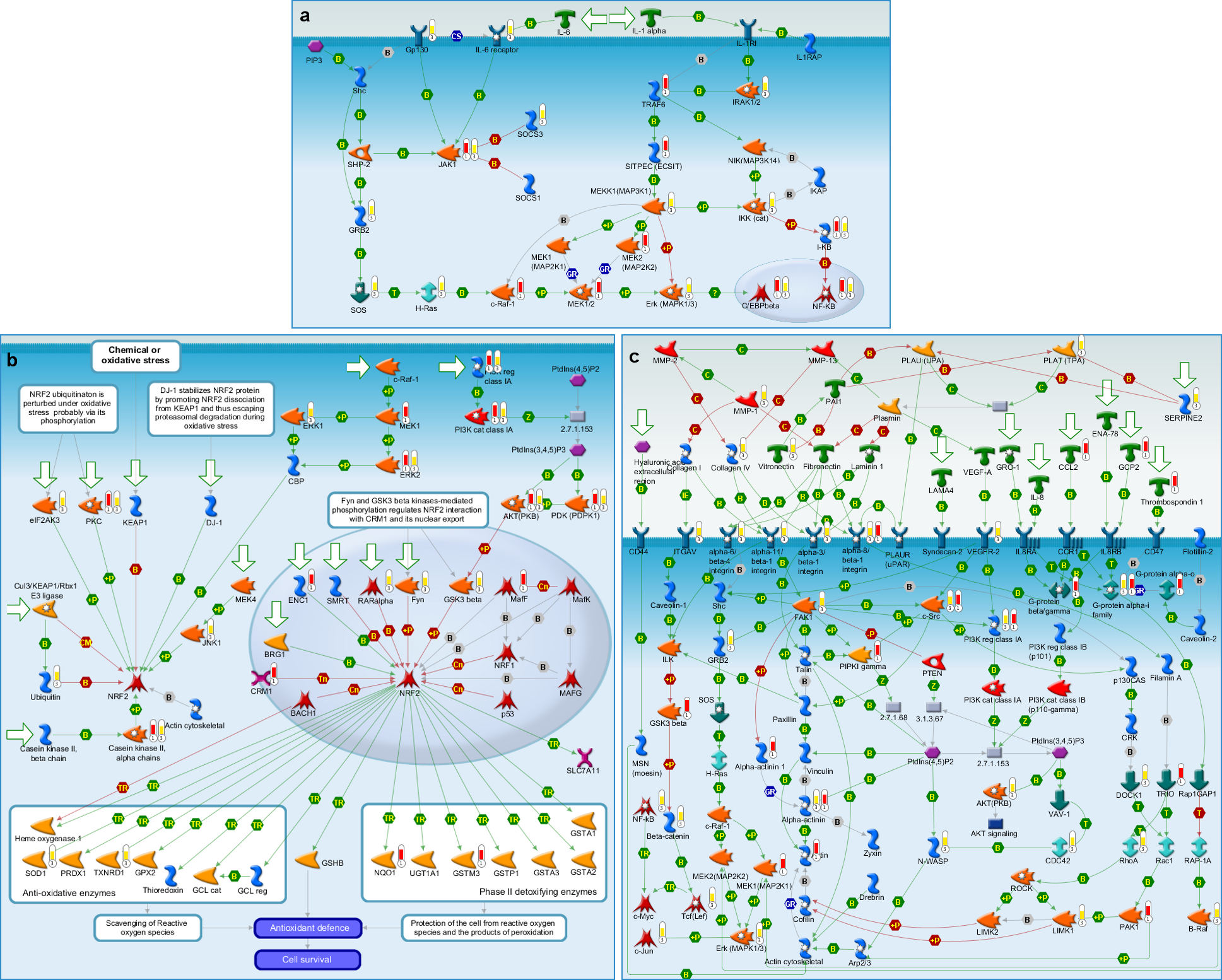
**

**Figure S3**

**Supplementary Figure S3 | Key pathways for different brain regions.** (a) *Signaling pathway mediated by IL-6 and IL-1* key pathway enriched with up-regulated (red) and topologically significant (yellow)

genes in s.nigra. (b) *Nrf2 regulation of oxidative stress response* key pathway enriched with up-regulated (red) and topologically significant (yellow) genes in striatum. (c) *Chemokines and adhesion* key pathway

enriched with up-regulated (red) and topologically significant (yellow) genes in cortex.

**
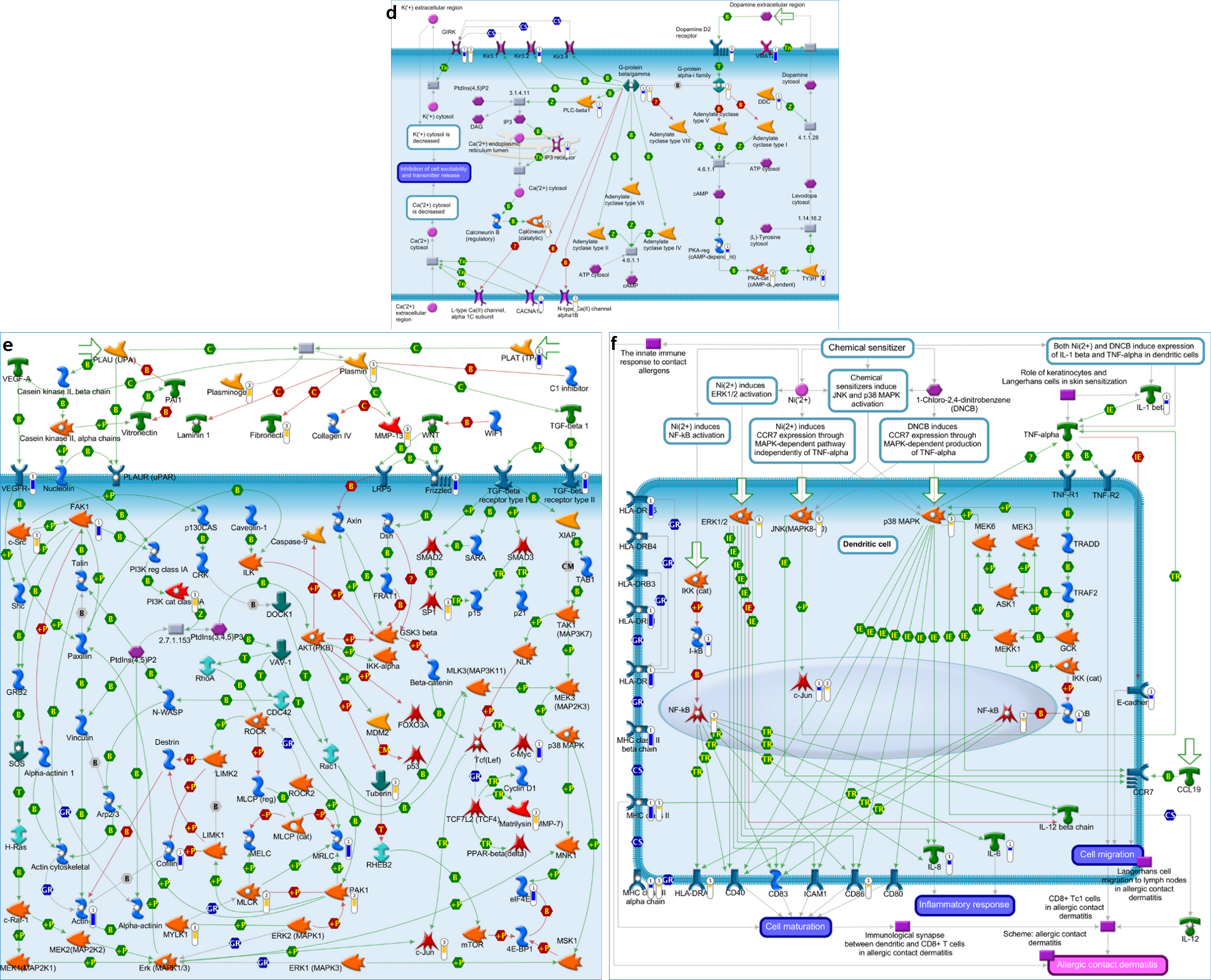
 Supplementary Figure S3| Key pathways for different brain regions.** (d) *Dopamine D2 receptor signaling in CNS* key pathway enriched with down-regulated (blue) and topologically significant (yellow)

**Figure S3**

genes in s.nigra. (e) *TGF, WNT and cytoskeletal remodeling* key pathway enriched with down-regulated (blue) and topologically significant (yellow) genes in striatum. (f) *Maturation and migration of dendritic cells*

*in skin sensitization* key pathway enriched with down-regulated (blue) and topologically significant (yellow) genes in cortex.

**
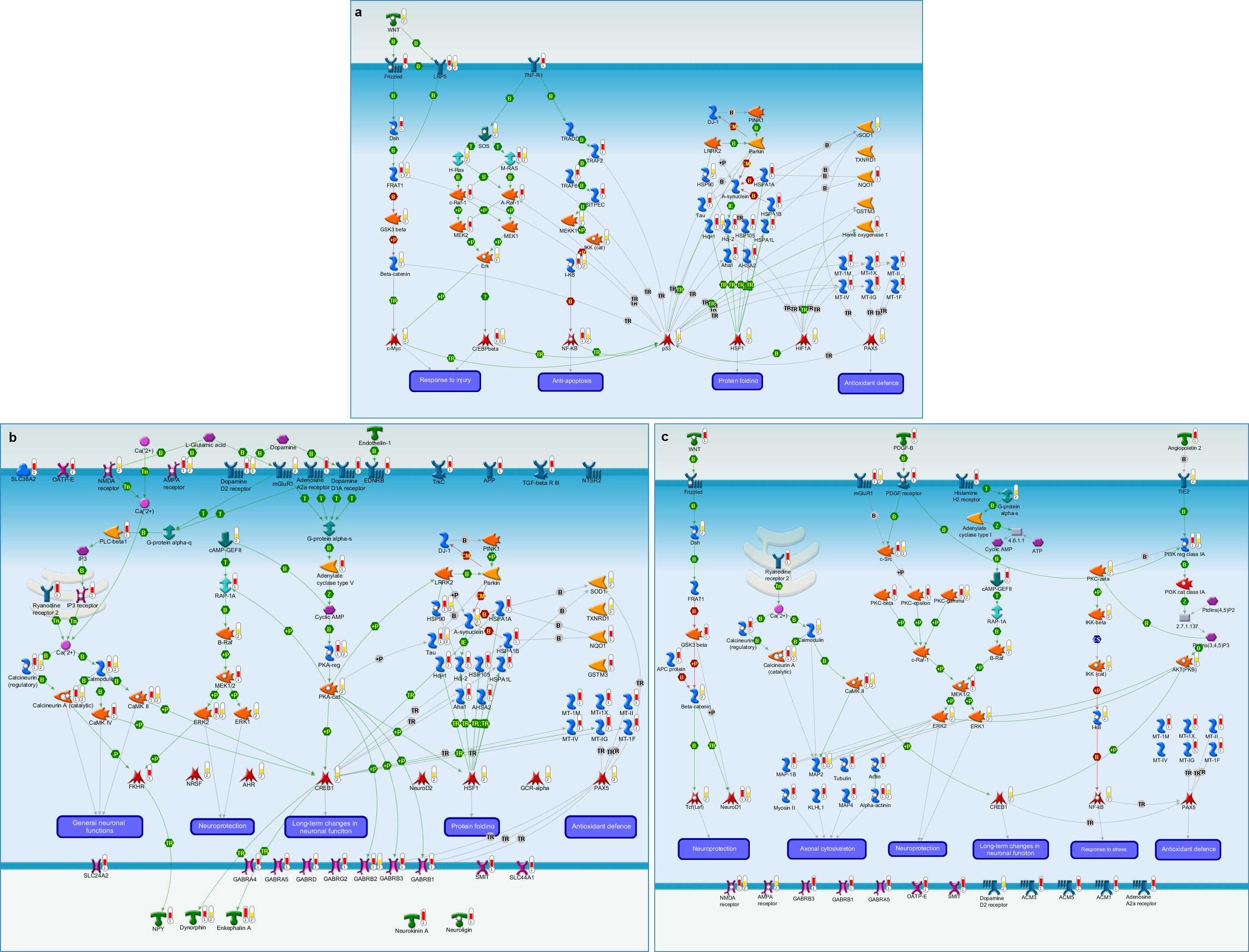
**

**Figure S4**

**Supplementary Figure S4 | Causal network models of individual brain regions.** (a) Causal network reconstruction based on up-regulated (red) and topologically significant (yellow) genes in s.nigra,

(b) Causal network reconstruction based on up-regulated (red) and topologically significant (yellow) genes in striatum. (c) Causal network reconstruction based on up-regulated (red) and topologically

significant (yellow) genes in cortex.


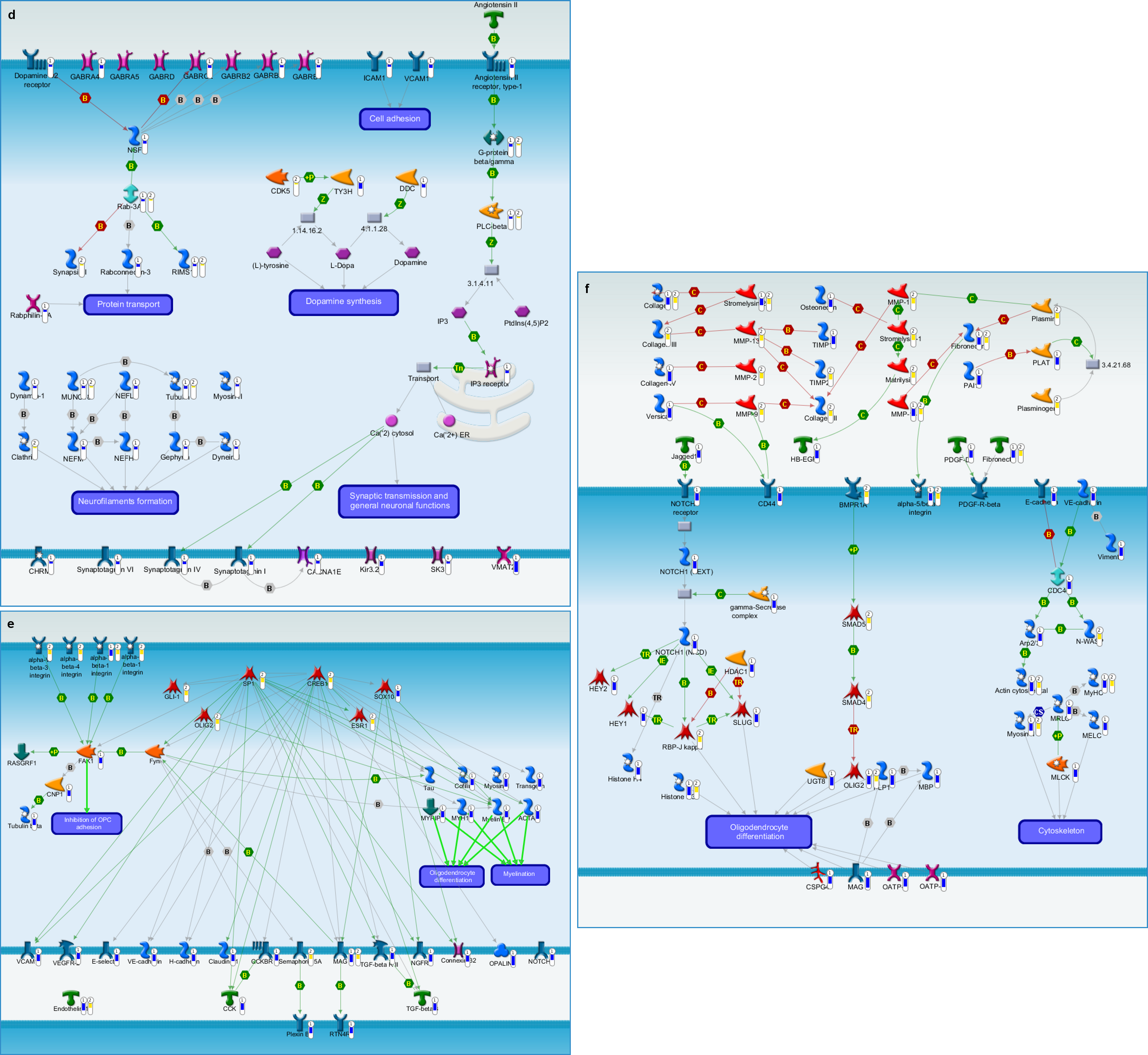


**Supplementary Figure S4 | Causal network models of individual brain regions.** (d) Causal network reconstruction based on down-regulated (blue) and topologically significant (yellow) genes in s.nigra. (e) Causal network reconstruction based on down -regulated (blue) and topologically significant (yellow) genes in striatum. (f) Causal network reconstruction based on down -regulated (blue) and topologically significant (yellow) genes in cortex.

**Figure S4**


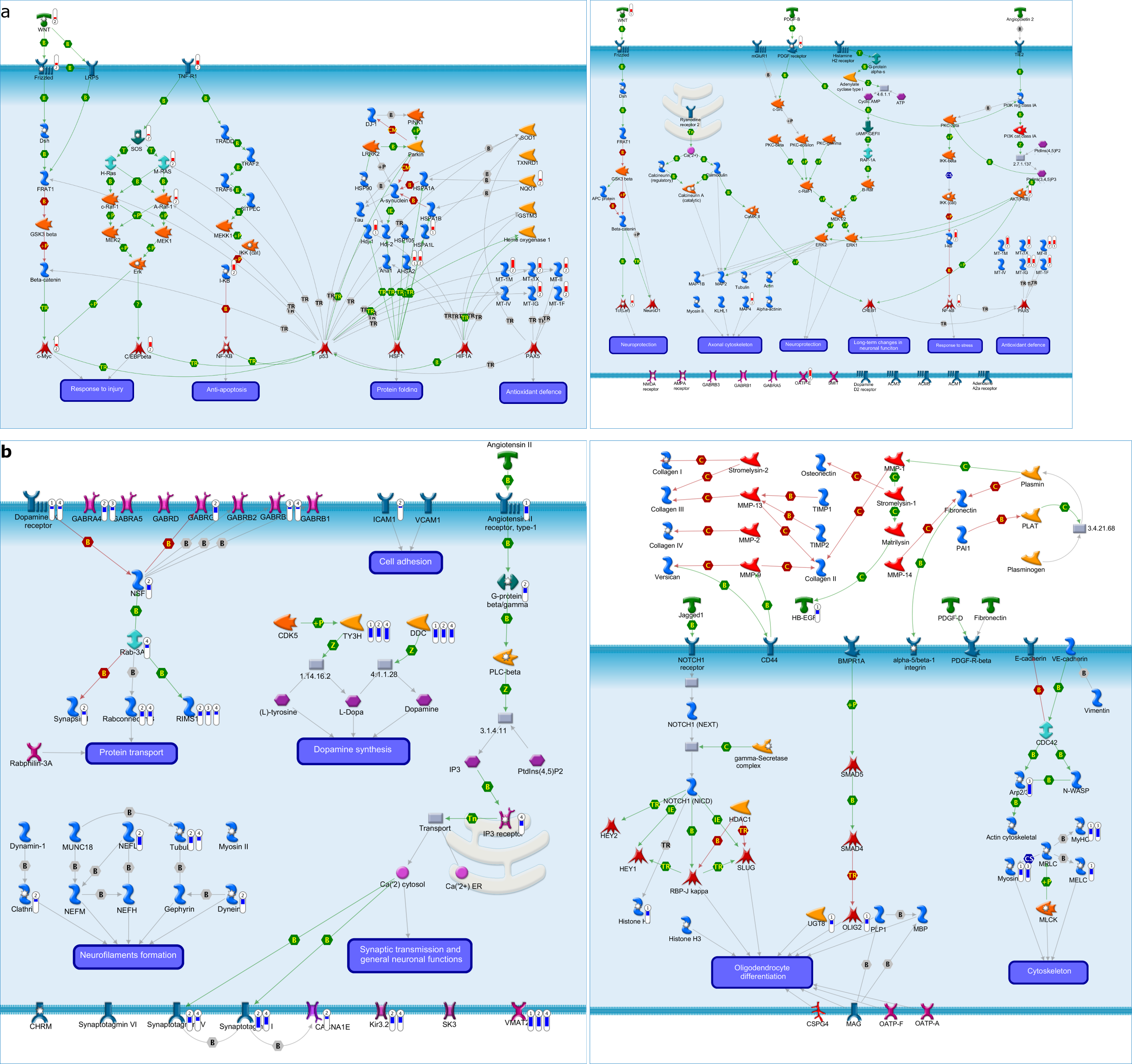


**Figure S5**

**Supplementary Figure S5 | Causal network models from different brain regions overlaid with ParkDB DEGs.** (a) Causal model overlaid with up-regulated genes (red) from

ParkDB for s.nigra on the left and cortex to the right. (b) Causal model overlaid with down-regulated genes (blue) from ParkDB for s.nigra on the left and cortex to the right.
