# Supplementary material for: Systems-Based Analyses of Brain Regions Functionally Impacted in Parkinson's Disease Reveals Underlying Causal Mechanisms
Source: PLoS One. 2014 Aug 29;9(8):e102909. doi: 10.1371/journal.pone.0102909 (PMC4149353; doi:10.1371/journal.pone.0102909)
Supplement: File S2 — Contains Figures S6–S12: Figure S6. Comparison of PD cortex, DLB cortex, and PD substantia nigra. Figure S7. Integrated causal network models from different brain regions. Figure S8. Enriched pathway maps and DEG overlaps in different brain regions identified by RNA-sequencing analysis. Figure S9. RNA-sequencing expression data overlaid onto causal networks. Figure S10. Overlap between proteomics and RNA-sequencing data. Figure S11. Enriched pathways and western blot validation of proteomics data. Figure S12. Alpha-synuclein levels correlated with peripheral biomarker levels. (DOC) [file pone.0102909.s012.doc]

**Figure S6**


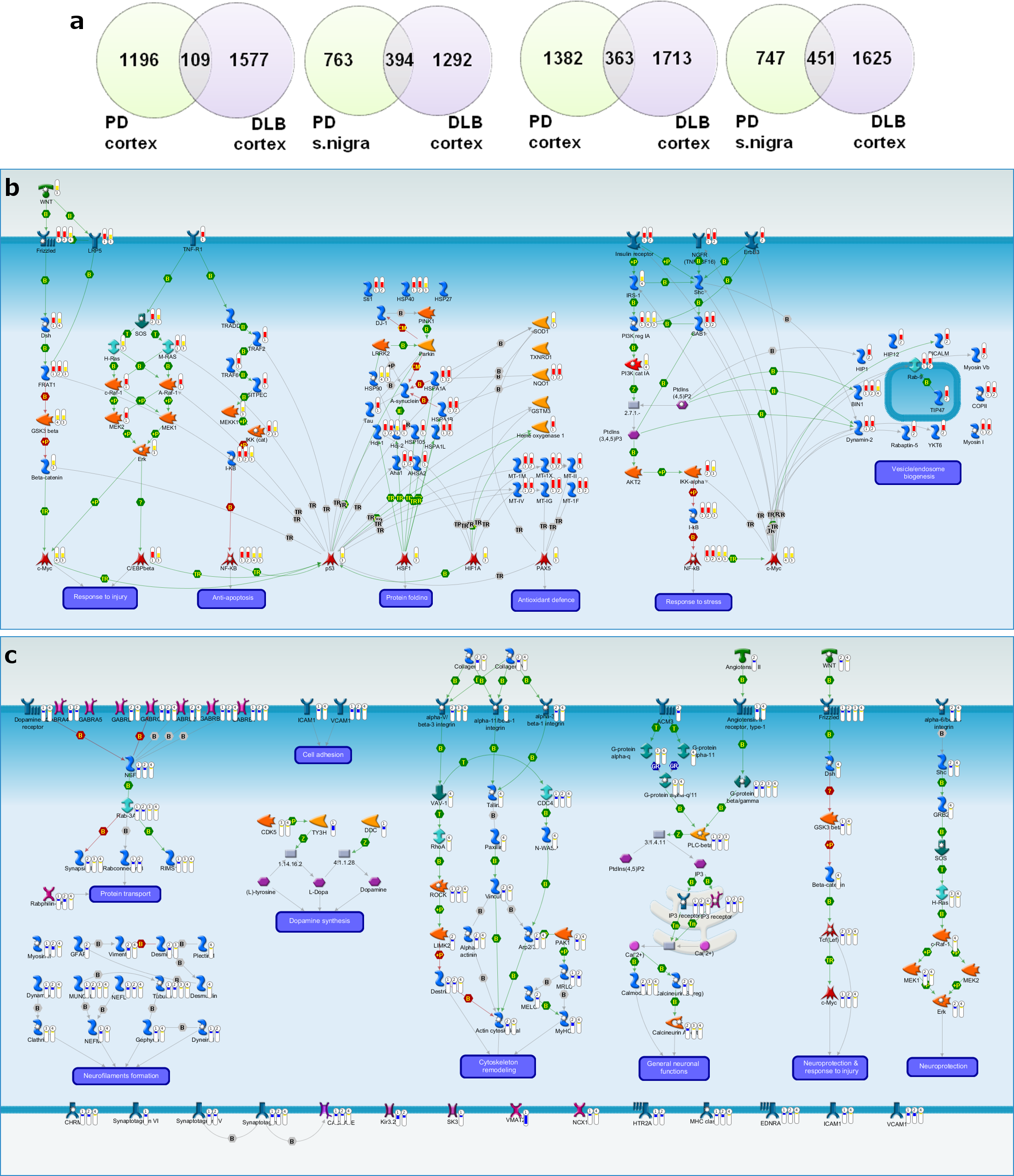


**Supplementary Figure S6 | Comparison of PD cortex, DLB cortex, and PD substantia nigra.** (a) Overlap at DEG level. Overlap between up-regulated genes are shown on the left, overlap between down-regulated genes are shown to the right. (b) Integrated causal network model for up-regulated genes in s. nigra (1) and DLB cortex (2) shown as red thermometers. Topologically significant genes for s.nigra (3) and DLB cortex (4) are shown as yellow thermometers. (c) Integrated causal network model for down-regulated genes in s. nigra (1) and DLB cortex (2) shown as blue thermometers. Topologically significant genes for s.nigra (3) and DLB cortex (4) are shown as yellow thermometer

**
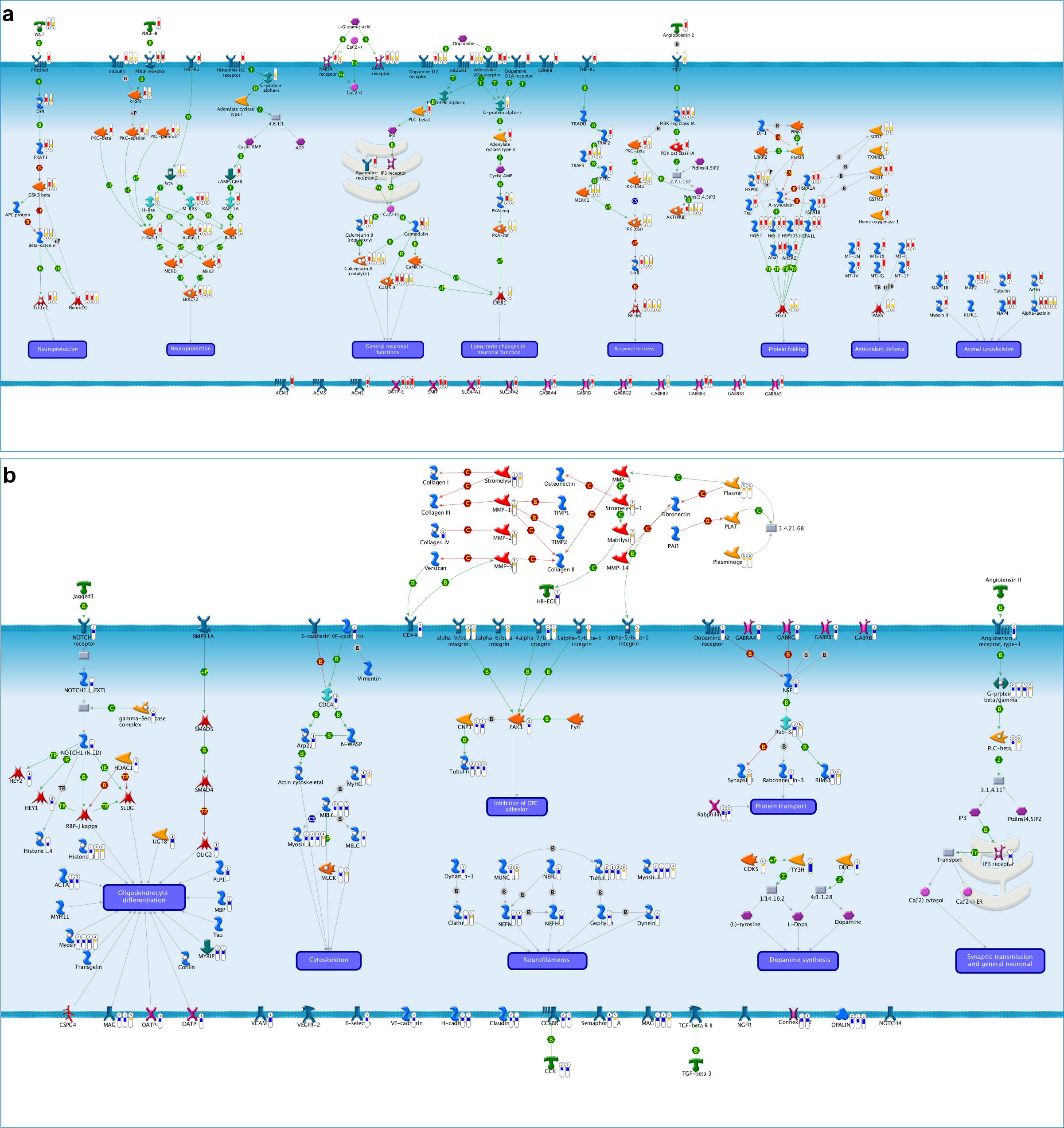
**

**Figure S7**

**Supplementary Figure S7 | Integrated causal network models from different brain regions.** (a) Integrated causal model overlaid with up-regulated genes (red) for cortex PD without up-regulated genes in DLB cortex (1), striatum PD without up-regulated genes in non-PD striatum (2), and s.nigra (3). Topologically significant genes for cortex PD (4), striatum PD (5) and s.nigra (6) are highlighted in yellow. (b) Integrated causal model overlaid with down-regulated genes (blue) for cortex PD without down-regulated genes in DLB cortex (1), striatum PD without down-regulated genes in non-PD striatum (2), and s.nigra (3). Topologically significant genes for cortex PD (4), striatum PD (5) and s.nigra (6) are highlighted in yellow.

**Figure S8**

**
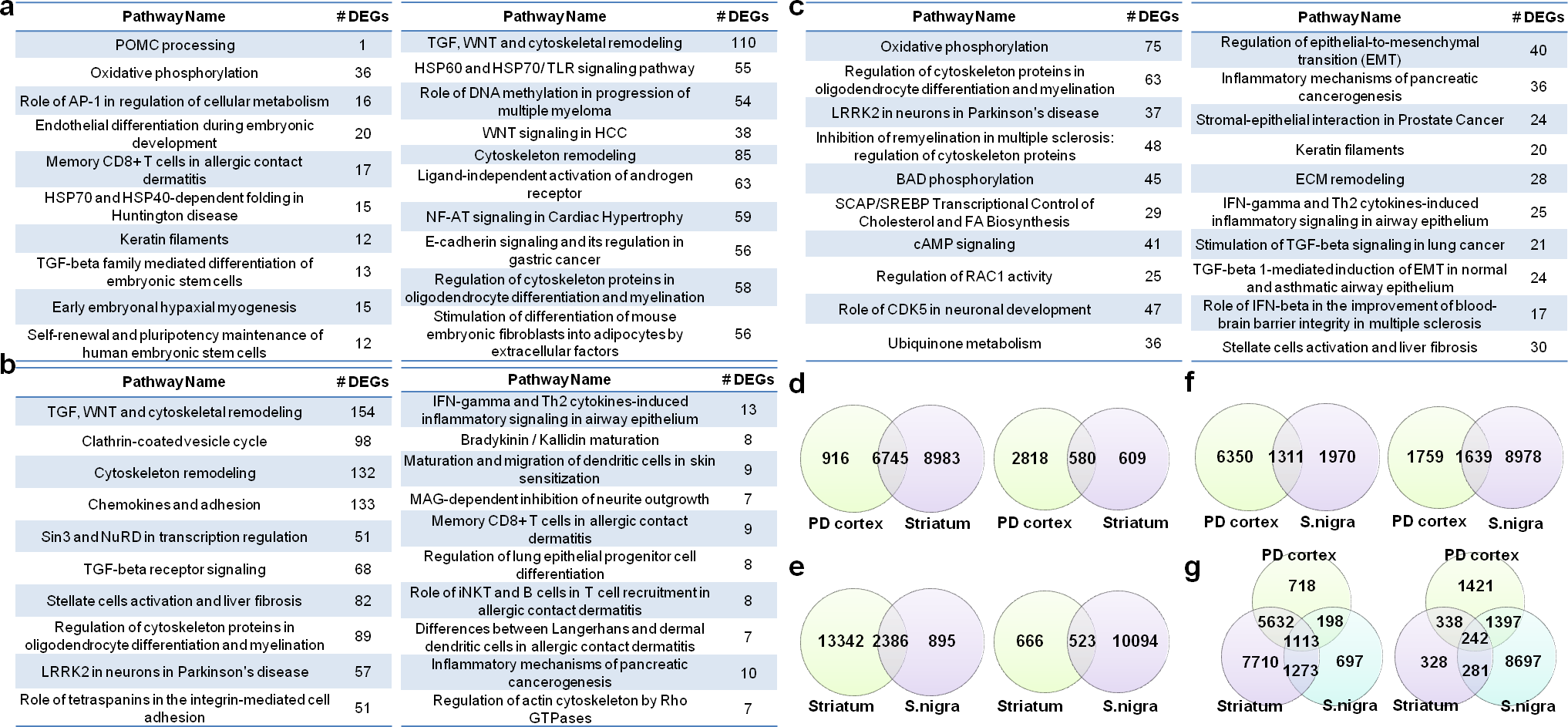
**

**Supplementary Figure S8 | Enriched pathway maps and DEG overlaps in different brain regions identified by RNA-sequencing analysis.** (a) Top 15 pathways enriched with RNAseq DEGs in s.nigra. Enrichments for up-regulated genes are shown on the left, for down-regulated genes on the right. (b) Top 15 pathways enriched with RNAseq DEGs in striatum. Enrichments for up-regulated genes are shown on the left, for down-regulated genes on the right. (c) Top 15 pathways enriched with RNAseq DEGs in cortex. Enrichments for up-regulated genes are shown on the left, for down-regulated genes on the right. (d) RNAseq DEG overlap between PD cortex and striatum. Overlap between up-regulated genes is shown on the left, down-regulated genes to the right. (e) RNAseq DEG overlap between striatum and s.nigra. Overlap between up-regulated genes is shown on the left, down-regulated genes to the right. (f) RNAseq DEG overlap between PD cortex and s.nigra. Overlap between up-regulated genes is shown on the left, down-regulated genes to the right. (g) RNAseq DEG overlap between the three brain regions. Overlap between up-regulated genes is shown on the left, down-regulated genes to the right.


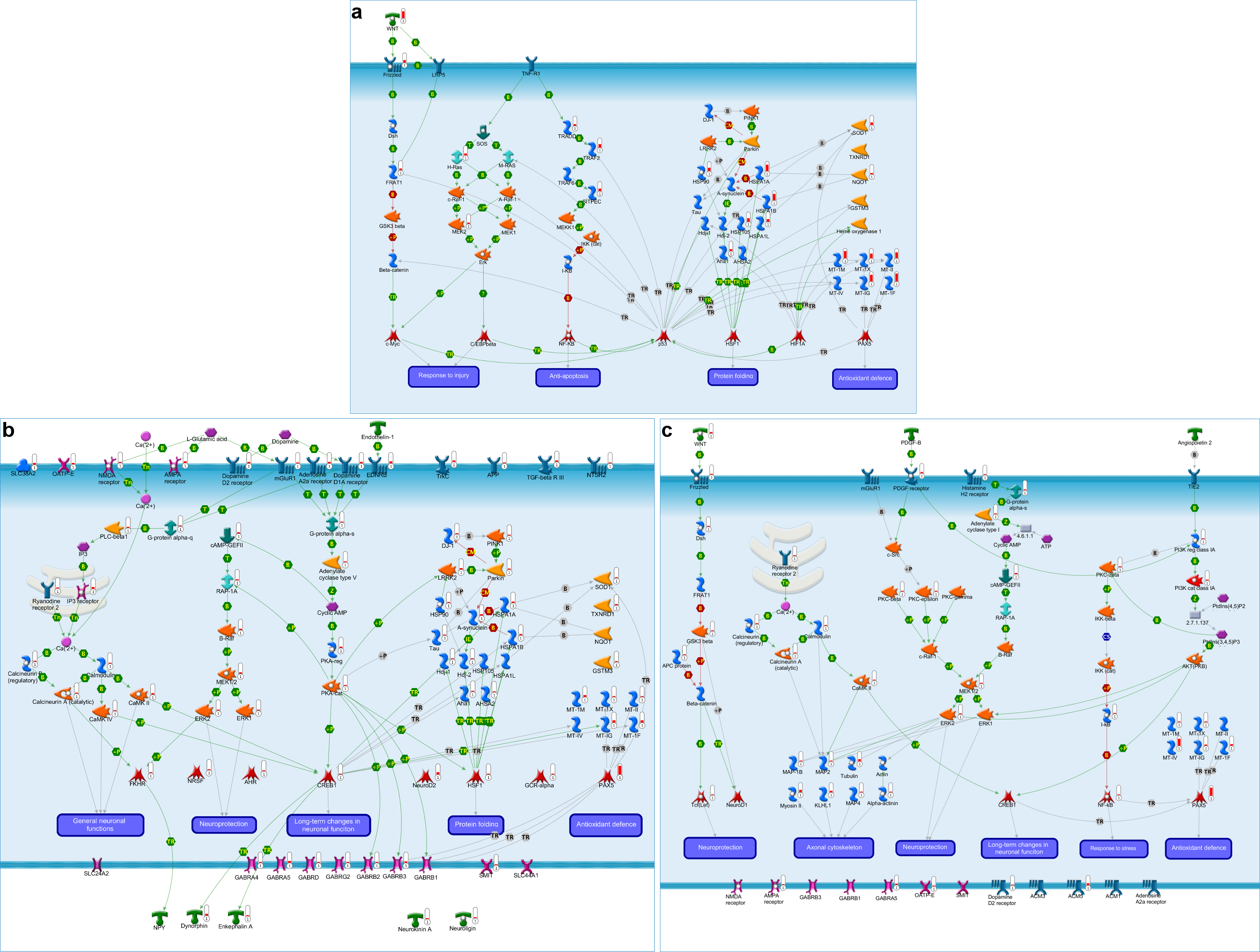
 **Figure S9**

**Supplementary Figure S9 | RNA-sequencing expression data overlaid onto causal networks.** (a) Causal network for s.nigra overlaid with up-regulated genes from RNA-seq analysis (red).

(B) Causal network for striatum overlaid with up-regulated genes from RNA-seq analysis (red). (c) Causal network for cortex overlaid with up-regulated genes from RNA-seq analysis (red).


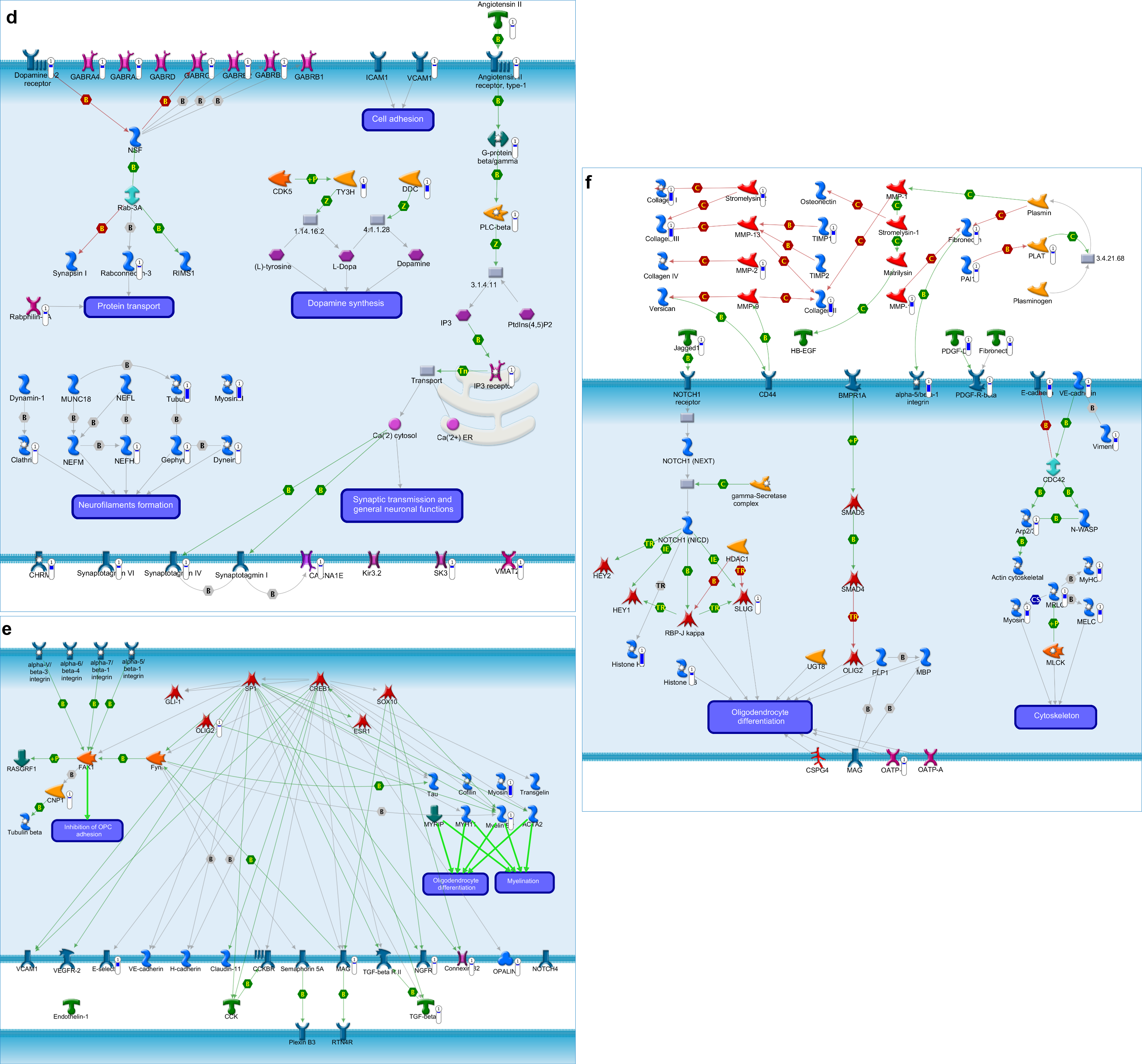


**Supplementary Figure S9 | RNA-sequencing expression data overlaid onto causal networks.** (d) Causal network for s.nigra overlaid with down-regulated genes from RNA-seq analysis (blue). (e) Causal network for striatum overlaid with down-regulated genes from RNA-seq analysis (blue). (f) Causal network for cortex overlaid with down-regulated genes from RNA-seq analysis (blue).

**Figure S9**

**Figure S10**

**
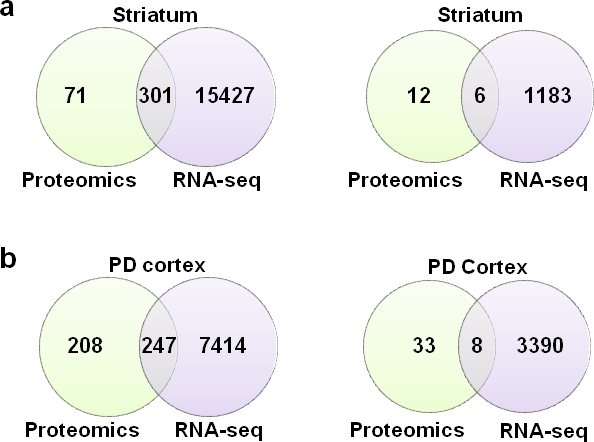
**

**Supplementary Figure S10 | Overlap between proteomics and RNA-sequencing data.** (a) Overlap in

striatum of proteins identified by proteomics and DEGs by RNA-seq analysis. Overlap of up-regulation is

shown on the left, down-regulation to the right. (b) Overlap in cortex of proteins identified by proteomics

and DEGs by RNA-seq analysis. Overlap of up-regulation is shown on the left, down-regulation to the right.

**Figure S11**

**
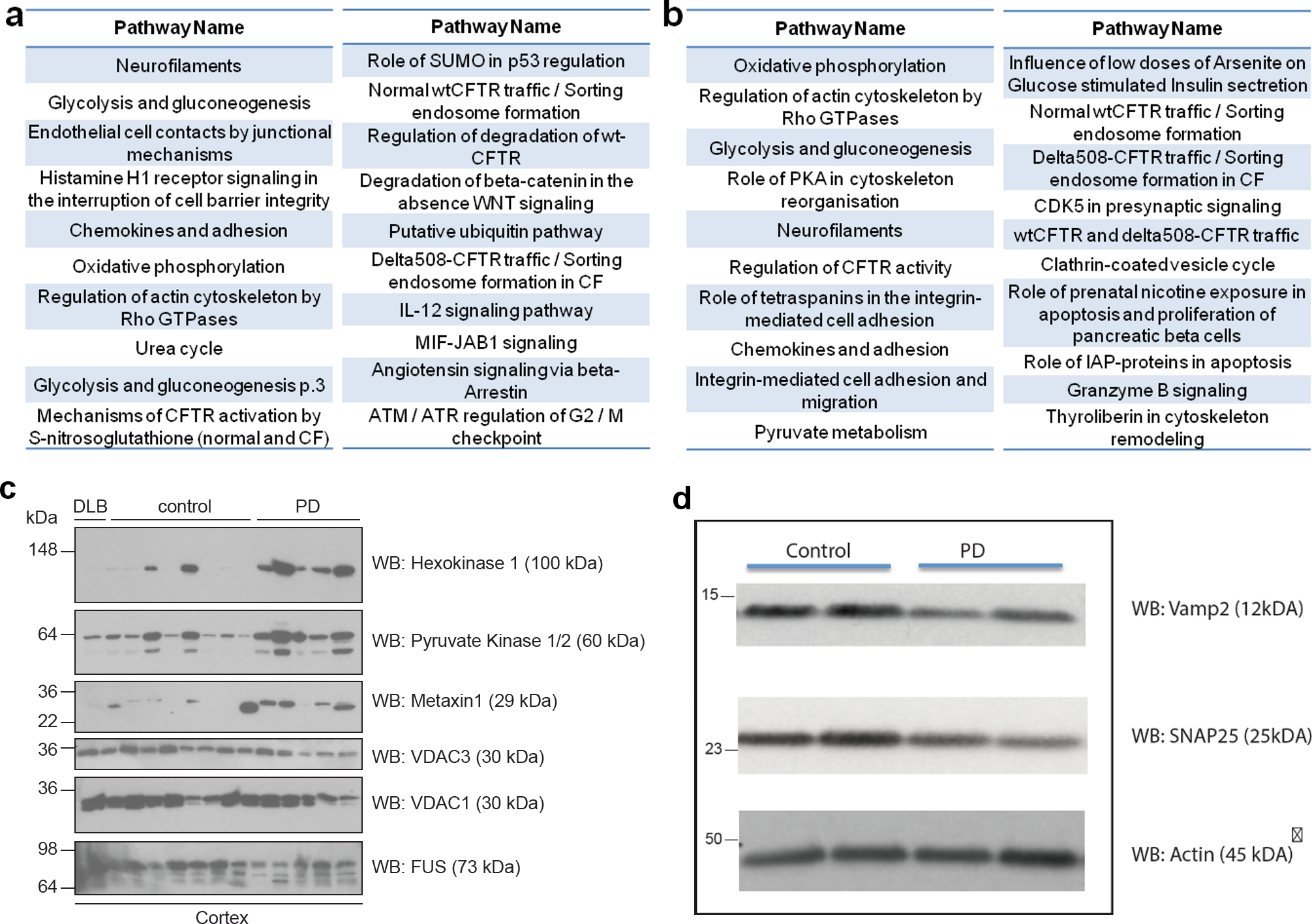
**

**Supplementary Figure S11 | Enriched pathways and western blot validation of proteomics data.** (a) Top pathways from striatum. Pathways enriched with increased protein levels are shown on the left and with decreased protein levels to the right. (b) Top pathways from cortex. Pathways enriched with increased protein levels are shown on the left and with decreased protein levels to the right. (c) Western blot validation of mass spectrometry data from cortex samples. Hexokinase 1, Pyruvate Kinase 1/2 and Metaxin 1 are all increased in PD samples compared to non-PD samples (control and DLB). VDAC1/3 and FUS levels are not significantly different between control and PD samples (d) Western blot validation of mass spectrometry data from striatum samples. VAMP and SNAP25 are decreased in PD samples compared to control.

**Figure S12**

**Supplementary Figure 12| Alpha-synuclein levels correlated with peripheral biomarker levels.** Correlation of cellular alpha-synuclein protein levels in peripheral white blood cells from control and PD patients with peripheral biomarker RNA levels was calculated using Pearson’s Correlation Coefficient (CAMK2B, r=0.7403; CXCR4, r=0.6468; AHSA2, r=-0.66112; ALDHA1, r=-0.3055). * p<0.05.
